# ZEB family is a prognostic biomarker and correlates with anoikis and immune infiltration in kidney renal clear cell carcinoma

**DOI:** 10.1186/s12920-024-01895-7

**Published:** 2024-06-05

**Authors:** Sheng Lin, Qi Chen, Canliang Tan, Manyi Su, Ling Min, Lv Ling, Junhao Zhou, Ting Zhu

**Affiliations:** 1 Department of Laboratory Medicine, Affiliated Cancer Hospital & Institute of Guangzhou Medical University, Guangzhou, Guangdong Province China; 2 Department of Urology, Foshan First People’s Hospital, Foshan City, Guangdong Province China; 3 Department of general surgery, The Third Affiliated Hospital of Southern Medical University, Guangzhou, Guangdong Province China; 4 KingMed school of Laboratory Medicine, Guangzhou Medical University, Guangzhou, Guangdong Province China; 5 Affiliated Cancer Hospital & Institute of Guangzhou Medical University, Guangzhou, Guangdong Province China

**Keywords:** ZEB family, Prognosis, Anoikis, Infiltrating immune cell, MicroRNAs, Kidney renal clear cell carcinoma

## Abstract

**Background:**

Zinc finger E-box binding homEeobox 1 (ZEB1) and ZEB2 are two anoikis-related transcription factors. The mRNA expressions of these two genes are significantly increased in kidney renal clear cell carcinoma (KIRC), which are associated with poor survival. Meanwhile, the mechanisms and clinical significance of ZEB1 and ZEB2 upregulation in KIRC remain unknown.

**Methods:**

Through the Cancer Genome Atlas (TCGA) database and Gene Expression Omnibus (GEO) database, expression profiles, prognostic value and receiver operating characteristic curves (ROCs) of ZEB1 and ZEB2 were evaluated. The correlations of ZEB1 and ZEB2 with anoikis were further assessed in TCGA-KIRC database. Next, miRTarBase, miRDB, and TargetScan were used to predict microRNAs targeting ZEB1 and ZEB2, and TCGA-KIRC database was utilized to discern differences in microRNAs and establish the association between microRNAs and ZEBs. TCGA, TIMER, TISIDB, and TISCH were used to analyze tumor immune infiltration.

**Results:**

It was found that ZEB1 and ZEB2 expression were related with histologic grade in KIRC patient. Kaplan-Meier survival analyses showed that KIRC patients with low ZEB1 or ZEB2 levels had a significantly lower survival rate. Meanwhile, ZEB1 and ZEB2 are closely related to anoikis and are regulated by microRNAs. We constructed a risk model using univariate Cox and LASSO regression analyses to identify two microRNAs (hsa-miR-130b-3p and hsa-miR-138-5p). Furthermore, ZEB1 and ZEB2 regulate immune cell invasion in KIRC tumor microenvironments.

**Conclusions:**

Anoikis, cytotoxic immune cell infiltration, and patient survival outcomes were correlated with ZEB1 and ZEB2 mRNA upregulation in KIRC. ZEB1 and ZEB2 are regulated by microRNAs.

**Supplementary Information:**

The online version contains supplementary material available at 10.1186/s12920-024-01895-7.

## Introduction

Renal cell carcinoma (RCC), which constitutes roughly 90% of renal malignancies and 2–3% of adult malignant diseases [1], is a neoplasm that arises from renal tubular epithelial cells [2]. Among the various histological subtypes of RCC, KIRC represents 70–80% of cases [3]. Due to its asymptomatic nature, a considerable proportion of KIRC patients, approximately 30%, are diagnosed at an advanced stage. The inherent resistance of advanced KIRC to radiate and chemical therapy underscores the importance of understanding the molecular mechanisms underlying early-stage diagnosis and prognostic analysis, as this knowledge can inform therapeutic strategies [4].

The preservation of tissue structure in human epithelial cells is achieved through cellular adhesion and interaction with the extracellular matrix [5]. Through anoikis, which is a form of programmed apoptosis, displaced cells within a tissue are eliminated as a result of a loss of cell-to-cell adhesion and matrix interaction [6]. Furthermore, Anoikis functions as a protective mechanism that impedes the metastasis of cancer cells. The survival of a cancer cell is reliant on its ability to resist anoikis through various signaling pathways, ultimately resulting in distant metastasis [7].The research has demonstrated that IL1RAP (IL-1 receptor accessory protein) plays a role in suppressing anoikis and impeding the spread of Ewing sarcoma. This is achieved by regulating the levels of cysteine and glutathione, thereby maintaining redox homeostasis and promoting resistance to anoikis [8]. Additionally, the downregulation of miR-30a in metastatic HCC promotes Beclin 1 and Atg5-dependent autophagy, leading to anoikis resistance [9].

Zinc finger E-box binding homeobox transcription factors (ZEBs) are a group of DNA-binding motifs found in eukaryotes, encompassing two distinct family members, specifically ZEB1 and ZEB2 [10]. The human ZEB1 gene is located on chromosome 10p11.22 and encodes a protein consisting of 1117 amino acids, while the human ZEB2 gene is situated on chromosome 2q22.3 and encodes a protein consisting of 1214 amino acids [11]. ZEB proteins are characterized by the presence of a centrally located homeodomain, along with several externally situated protein binding domains including the zinc finger domain, SMAD interaction domain, p300-CBP-associated factor binding domain, CtBP interaction domain, and coactivator binding domain. These domains play a crucial role in the regulation of tumor metastasis and progression through the process of epithelial-mesenchymal transition (EMT) [12].

There is a wealth of evidence indicating that immune infiltration is a crucial factor in the development and advancement of both oncogenesis and cancer progression. The microenvironment of KIRC, a highly immunogenic cancer, is distinguished by a significant presence of T cells and other immune cells [13]. To further explore the relationship between ZEB1/2 and immune infiltration in KIRC, we conducted an extensive investigation utilizing The Tumor and Immune System Interaction Database (TISIDB), The Tumor Immune Estimation Resource (TIMER2.0), and The Tumor Immune Single-cell Hub (TISCH).

**Fig. 1:**
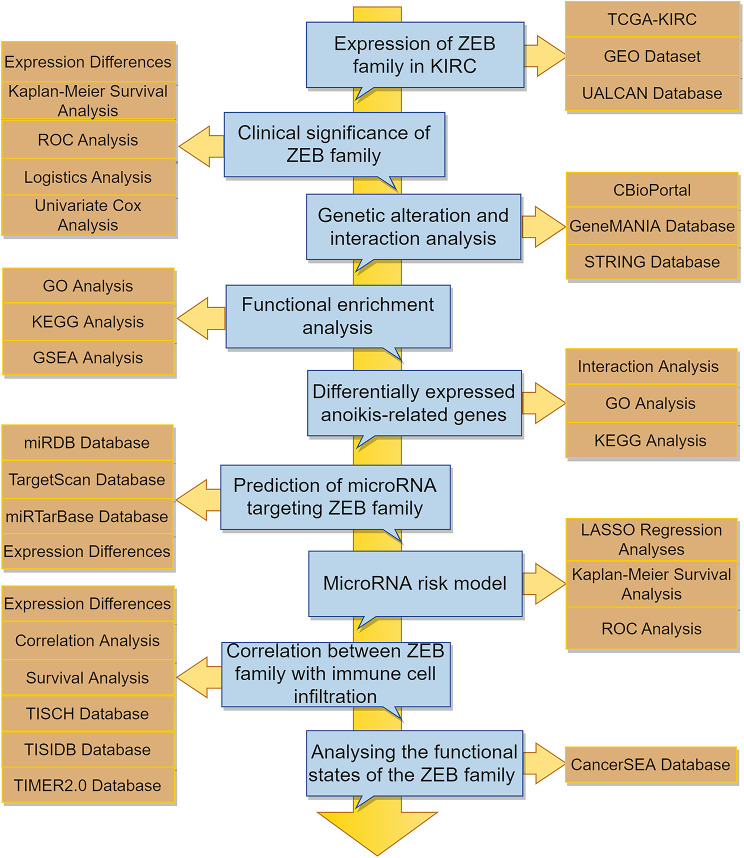
Article flowchart. The expression of ZEB1 and ZEB2 in KIRC is correlated with unfavorable prognosis. The flow chart comprehensively illustrates the various analyses conducted in this study. It is observed that microRNAs (hsa-miR-130b-3p and hsa-miR-138-5p) play a regulatory role in modulating the expression of ZEB1 and ZEB2. The upregulation of ZEB1 and ZEB2 mRNA in KIRC significantly affects anoikis, immune cell infiltration, and patient survival. These findings offer valuable insights into the molecular mechanisms and clinical relevance of ZEB1 and ZEB2 in the context of KIRC

To elucidate the mechanism of ZEB1 and ZEB2 in KIRC, we integrated multiple bioinformatics databases. Our study systematically delineated the roles of ZEB1 and ZEB2 in the progression, metastasis, and clinical prognosis of KIRC. Notably, ZEB1 and ZEB2 exert pleiotropic effects on various metabolic pathways, underscoring their significance in KIRC. This flow chart describes the study’s procedures (Fig. 1).

## Materials and methods

### The analysis using the cancer genome atlas (TCGA)

Over 20,000 matched tumor samples and normal samples were included in TCGA, an important cancer genomics initiative for identifying more than 33 cancer types (http://cancergenome.nih.gov/).

### The analysis using GEO

In 2000, the National Center for Biotechnology Information created the GEO database (https://www.ncbi.nlm.nih.gov/) to store high-throughput gene expression data. Scientists from around the world upload various types of high-throughput genomics data, such as microarrays and next-generation sequencings, to the GEO database. This database effectively collects and organizes data pertaining to both tumor and non-tumor diseases. Relevant data was obtained by searching the GEO database. We obtained the GSE40435 dataset from the GEO repository and performed differential analysis using R packages: ggplot2 [3.3.6], stats [4.2.1], and car [3.1-0]. In the GSE40435 dataset, there were 101 sets of ccRCC tumors and neighboring non-tumor renal tissue. In addition, WGCNA analysis was performed on the GSE66270 dataset with 14 groups of ccRCC tumors and adjacent non-tumor kidney tissue.

#### XIANTAO platform

The XIANTAO platform, accessible at https://www.xiantaozi.com/, serves as a comprehensive database that consolidates TCGA tumor microarray data along with R software and its associated packages. This platform is predominantly utilized for conducting research on gene expression, correlation, enrichment, interaction networks, clinical significance, and localization analysis. The XIANTAO was employed to examine the expression of ZEB1 and ZEB2, as well as the correlations between ZEB1, ZEB2, and various clinicopathological indices of TCGA-KIRC. To assess the overall survival (OS), disease-specific survival (DSS), and progress-free interval (PFI) using hazard ratio (HR) with 95% confidence intervals (CIs) and log-rank P values, the patient samples were stratified into two groups based on the median expression levels of ZEB1 and ZEB2 (high expression vs. low expression). Using the XIANTAO platform, we evaluated the forecast-worth of ZEB1 and ZEB2 in KIRC.

The identification of co-expressed genes of ZEB1 and ZEB2 was accomplished by employing spearman correlation coefficients (*p* < 0.001, *r* > 0.7). Subsequently, the clusterProfiler package was utilized to perform Kyoto Encyclopedia of Genes and Genome (KEGG) and Gene ontology (GO) analyses, aiming to explore the potential signal pathways and biological functions regulated by ZEB1 and ZEB2. The gene ontology analysis encompassed the categorizations of molecular function (MF), cellular component (CC), and biological process (BP). The TCGA gene expression data was analyzed using GSEA.A false discovery rate (FDR) < 0.25 and a p.adjustment < 0.05 were the criteria used to determine significant enrichment.

We conducted a study utilizing the XIANTAO resource to examine the infiltration patterns of the immune system within tumors. Various immunocytes were identified by employing a combination of 24 immunological markers. The Spearman correlations between immunocyte biomarkers and the expression levels of ZEB1 and ZEB2 were calculated using the single-sample GSEA (ssGSEA) method.

#### Weighted gene co-expression network analysis (WGCNA)

WGCNA facilitates the extraction of biologically relevant module information through the examination of pairwise correlations among genes in high-throughput data, utilizing the WGCNA package [14, 15]. The co-expression network was established by selecting the top 25% of genes with the greatest expression variance, and adjacency matrices were computed based on Pearson’s correlation coefficients to store comprehensive information on the entire co-expression network. The average linkage hierarchical clustering method was utilized to cluster dendrograms with a minimum module size of 20, using topological overlap measure (TOM) matrices. Subsequently, gene modules exhibiting similarity were merged with a threshold of 0.25, and the significantly distinct modules in tumor tissues as compared to normal tissues were analyzed to identify key genes.

#### The University of Alabama at Birmingham cancer data analysis portal (UALCAN)

Researchers have the ability to thoroughly analyze transcriptome data from the TCGA, MET500, and Clinical Proteomic Tumor Analysis Consortium using UALCAN (http://ualcan.path.uab.edu/analysis.html).UALCAN was utilized in KIRC to examine the protein composition and levels of promoter methylation in ZEB1 and ZEB2.

#### A web of genes that interact with ZEB1 and ZEB2, as well as protein-protein interactions (PPIs)

The GeneMANIA database (http://www.genemania.org/) was employed to construct the ZEB1 and ZEB2 gene-gene interaction networks, thereby generating hypotheses regarding gene function and identifying genes with comparable functions. Additionally, the STRING database (https://string-db.org/) was utilized to construct, visualize, and analyze the protein-protein interaction networks associated with ZEB1 and ZEB2.

### CBioPortal

The cBioPortal for Cancer Genomics (https://www.cbioportal.org/) enables the visualization, abstraction, and assessment of extensive cancer genomics datasets. Mutations in ZEB1 and ZEB2 were examined in 1496 KIRC patients sourced from the cBioPortal database.

### Acquisition of data and screening of anoikis-related genes

By using GeneCards database (https://www.genecards.org/), 338 genes associated with anoikis were selected, and only relevance score exceeding 1.0 genes were considered [16]. Further analyses were conducted by combining ZEB1 and ZEB2 with the selected differentially expressed genes related to anoikis in KIRC.

### Resource for estimating immune response in tumors

TIMER2.0 online tool (http://timer.cistrome.org/) was used to analyze immune infiltrates in different cancer type. In this study, the Exploration-Gene DE module of TIMER2.0 was used to evaluate the expressions of ZEB1 and ZEB2 in various malignancies. Specifically, in the KIRC dataset, TIMER2.0 was employed to investigate the relationship between ZEB1 and ZEB2 expressions and the infiltration of immune cells. The “Immune-Gene” module was investigated the correlation between ZEB1, ZEB2 and the invasion levels of immune cells (CD4 + T cells, CD8 + T cells, B cells, neutrophils, dendritic cells, and macrophages).

### Database for the interaction between tumors and the immune system (TISIDB)

The TISIDB (http://cis.hku.hk/TISIDB/index.php) holds comprehensive information on the relationship between the immune system and tumors. The ZEB1 and ZEB2 interactions with T-cell checkpoints were examined in the ‘Immunomodulator’ module using Spearman’s correlation coefficient.

### Analysis of the Kaplan-Meier plotter database

The KM Plotter data resource (https://kmplot.com/analysis/) contains collection of immune cell infiltration data, survival information and gene expression data from 530 patients with KIRC. The information from particular data source was utilized to evaluate predictions for associated immune cell subcategories depending on the presence of ZEB1 or ZEB2 in KIRC.

### Levels of ZEB1 and ZEB2 expression in individual cells

Tumor immune single-cell hub (TISCH) offers an interactive visualization of tumor microenvironments through its website (http://tisch.comp-genomics.org/home/) [17]. For instance, we employed the dataset module to visualize the expression levels of ZEB1 and ZEB2 at the single-cell level in the KIRC_GSE111360 and KIRC_GSE139555 datasets, respectively [18, 19].

### Single-cell function in cancer

The Cancer Single-cell Atlas (CancerSEA) database (http://biocc.hrbmu.edu.cn/CancerSEA/) allows the functional state of specific genes to be observed at the single-cell level, thereby overcoming the limitations of traditional approaches to gene expression analysis [20].

### MiRNA prediction

The current research employed a fairly extensive approach for miRNA prediction. To predict targeted miRNAs of mRNAs, three databases were utilized: miRDB (http://www.mirdb.org/), TargetScan (https://www.targetscan.org/), and miRTarBase (https://www.mirbase.org/).

### Statistical analysis

For non-paired samples, the Shapiro-Wilk test is used to check the normality of the data. If the data follows a normal distribution, the Student’s t-test is used to assess the differences between the two groups; if the data does not follow a normal distribution, a rank sum test is used for comparison. In addition, for paired samples, either a paired t-test or Wilcoxon signed-rank test is used, with the Shapiro-Wilk test employed to examine the normality assumption of the paired differences. A P-value less than 0.05 is considered statistically significant. A significant difference was considered when the P value was below 0.05 in all analyses. To perform the analysis, the analysis was conducted using the R project website (https://www.r-project.org/) and the R online tool.

## Results

### Variations in the expression levels of ZEB1 and ZEB2 among patients diagnosed with KIRC

**Fig. 2:**
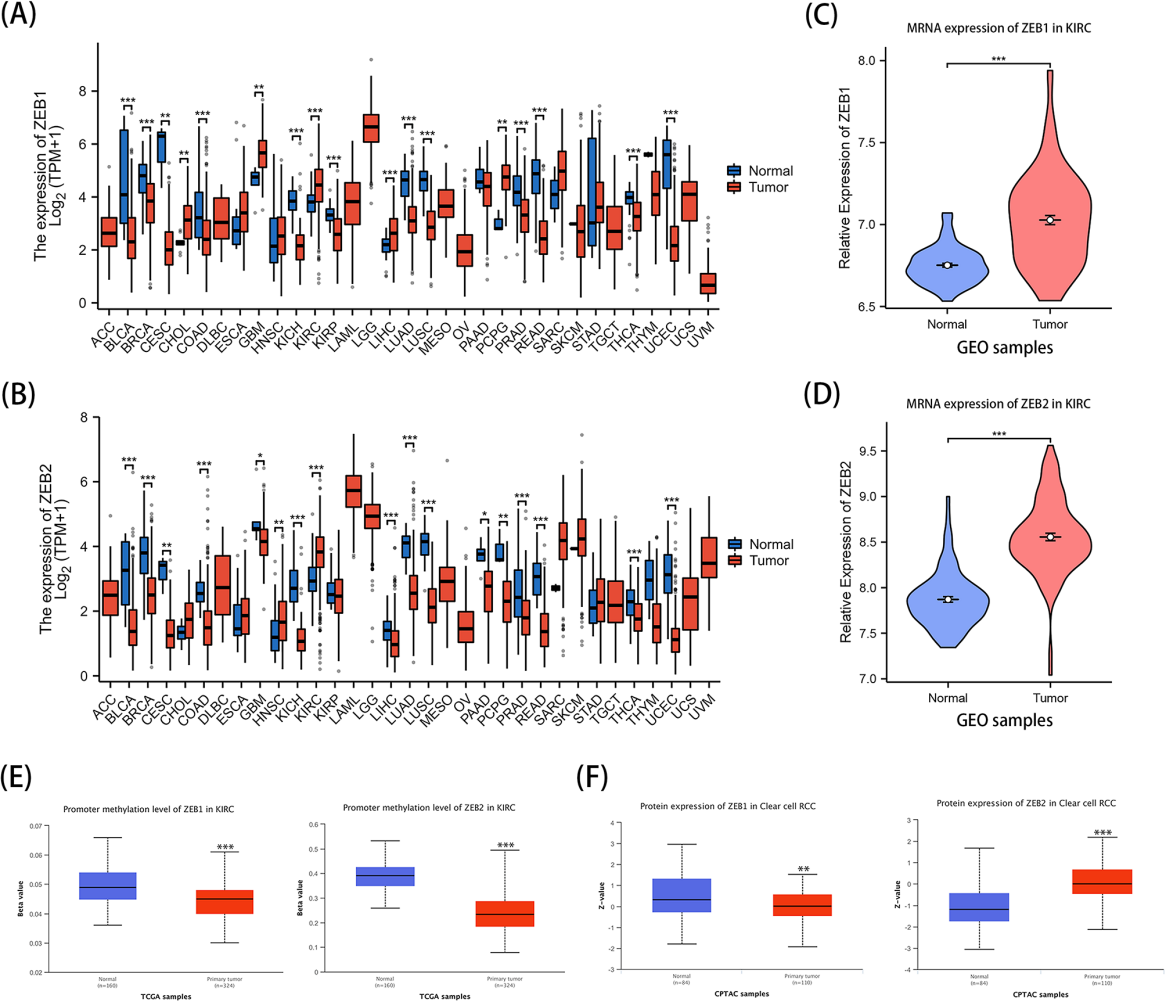
Expression of ZEB1 and ZEB2 in Kidney Renal Clear Cell Carcinoma (KIRC). A, ZEB1 expression in various types of cancer was determined using the Tumor Immune Estimation Resource (TIMER) database. B, ZEB2 expression in different types of cancer. C, D, Expression levels of ZEB1 (C) and ZEB2 (D) were evaluated in KIRCs and their corresponding adjacent normal tissues using the GSE40435 dataset. E, Promoter methylation levels of ZEB1 and ZEB2 in KIRC tissues and normal tissues were analyzed using UALCAN. F, The UALCAN database was utilized to examine the protein expressions of ZEB1 and ZEB2 in KIRC. Note: *P < 0.05; **P < 0.01; ***P < 0.001

The expression of ZEB1 and ZEB2 in solid cancer of humans was initially evaluated using the TCGA data resource. The findings, illustrated in Fig. 2A and B, suggest that ZEB1 and ZEB2 exhibit significant expression levels in KIRC. Supplementary Fig. 1A, B provide additional evidence of the pan-cancer expression of ZEB1 and ZEB2 in both tumor and normal tissues. Additionally, we performed a comparative examination of the expressions of ZEB1 and ZEB2 in KIRC samples and neighboring non-tumorous renal tissue using GSE40435. This analysis unveiled a noteworthy increase in the expression of both genes (Fig. 2C, D). Furthermore, the UALCAN data source exhibited a notable decrease in the methylation levels of ZEB1 and ZEB2 promoters in tumor tissues when compared to normal tissues (Fig. 2E). Moreover, the analysis of the UALCAN database indicated a significant decline in the expression of ZEB1 protein in KIRC tissues when compared to healthy tissues. This was accompanied by a marked rise in the expression of ZEB2 protein, as shown in Fig. 2F. Furthermore, elevated levels of ZEB1 and ZEB2 were observed in both tumor and normal tissues (Supplementary Fig. 1). The WGCNA analysis of GSE66270 revealed a positive correlation between the blue and red modules and tumor discovery (Supplementary Fig. 2). ZEB1 is a member of the red module, while ZEB2 is associated with the blue module (Supplementary Fig. 2C). Additionally, ZEB1 plays a significant role within the red module (Supplementary Fig. 2D). The results indicate that ZEB1 and ZEB2 might have significant functions in the advancement of KIRC.

### Examining the correlation between the expressions of ZEB1 and ZEB2 and the clinical parameters of patients diagnosed with KIRC

**Fig. 3:**
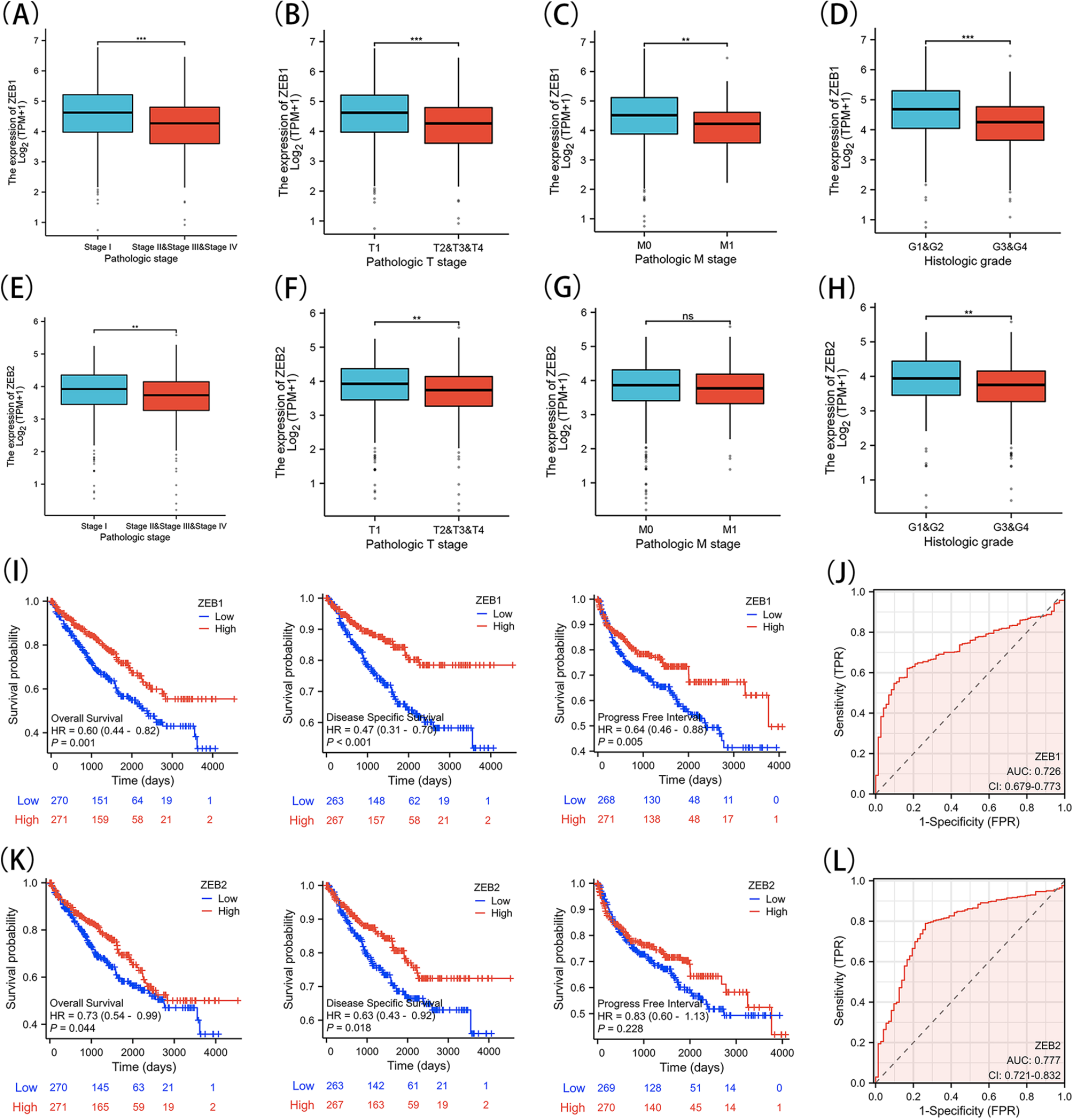
Relationship between Gene Expression and Clinical Characteristics, Kaplan-Meier Survival Analysis and ROC analysis. A, E, Pathologic stage of ZEB1 and ZEB2. B, F, respectively showing the tumor stage of ZEB1 and ZEB2 expressions in KIRC. C, G, metastasis stage of ZEB1 and ZEB2. D, H Histologic grade of ZEB1 and ZEB2 expressions. I, Survival curves of overall survival (OS), disease-specific survival (DSS), and progress-free interval (PFI) between ZEB1-high and -low patients with KIRC. K, OS, DSS, and PFI between ZEB2-high and -low patients with KIRC. J, L, Receiver operating characteristic curve of ZEBs in diagnosis of KIRC. Note: ns (not significant); **P < 0.01; ***P < 0.001

We utilized the XIANTAO platform’s online tool to examine the unique manifestations of ZEB1 and ZEB2 in various patient groups categorized by clinical parameters (Supplementary Tables 1, 2). Significantly lower expression levels of ZEB1 and ZEB2 were observed in patients with pathologic stages II, III, and IV in comparison to those with stage I, as indicated by our analysis of KIRC patients categorized by their pathologic stage (Fig. 3A and E). The levels of ZEB1 and ZEB2 were notably decreased in KIRC patients with T2, T3, and T4 stages in comparison to those with T1 stage, as depicted in Fig. 3B and F. During the M0 stages, it was observed that the level of ZEB1 expression was greater compared to the M1 stages (Fig. 3C), whereas the expression of ZEB2 remained unchanged (Fig. 3G). In addition, patients with G3 and G4 stages exhibited a notable reduction in the expression of ZEB1 and ZEB2, as observed in the histologic grade of KIRC, in contrast to those with G1 and G2 stages (Fig. 3D and H). The evidence presented suggests that ZEB1 and ZEB2 might have important functions in the initial phases of KIRC formation and influence the entire progression of KIRC expansion.

### A decreased ZEB1 and ZEB2 mRNA level is associated with poor prognosis in KIRC

The above-mentioned results suggest a link between ZEB1 and ZEB2 and the advancement of KIRC. As a result, we proceeded to examine their predictive importance in KIRC (Tables 1 and 2). By analyzing the TCGA database, we found that decreased ZEB1 and ZEB2 expression levels were associated with worse overall survival (OS), disease-specific survival (DSS), and progress-free interval (PFI) (Fig. 3I, K). The results indicate that lower amounts of ZEB1 and ZEB2 are associated with an adverse prognosis in patients with KIRC. Following that, we performed Cox univariate analyses to validate the importance of ZEB1, ZEB2, and various clinical factors including age, sex, ethnicity, TNM stage, pathological stage, histological stage, primary therapy outcome, laterality, and hemoglobin levels (Supplementary Fig. 3). Furthermore, the AUC values of KIRC were 0.726 and 0.777 (Fig. 3J, L).

**Table 1:** ZEB1 single-gene logistic regression

Characteristics	Total (*N*)	OR (95% CI)	*P* value
Pathologic T stage (T3&T4&T2 vs. T1)	532	0.513 (0.363–0.724)	< 0.001
Pathologic N stage (N1 vs. N0)	256	0.600 (0.211–1.703)	0.337
Pathologic M stage (M1 vs. M0)	500	0.457 (0.276–0.755)	0.002
Pathologic stage (Stage III&Stage IV&Stage II vs. Stage I)	529	0.507 (0.359–0.716)	< 0.001
Primary therapy outcome (PR&CR vs. PD&SD)	138	1.259 (0.444–3.574)	0.665
Gender (Male vs. Female)	532	0.730 (0.511–1.044)	0.085
Race (White vs. Asian&Black or African American)	525	1.233 (0.730–2.085)	0.434
Age (> 60 vs. <= 60)	532	0.718 (0.510–1.010)	0.057
Histologic grade (G3&G4 vs. G1&G2)	524	0.430 (0.302–0.611)	< 0.001
Serum calcium (Normal vs. Low&Elevated)	364	0.596 (0.390–0.909)	0.016
Hemoglobin (Normal vs. Low&Elevated)	452	0.971 (0.667–1.413)	0.876
Laterality (Right vs. Left)	531	1.137 (0.808–1.599)	0.462

**Table 2:** ZEB2 single-gene logistics regression

Characteristics	Total (*N*)	OR (95% CI)	*P* value
Pathologic T stage (T3&T4&T2 vs. T1)	532	0.616 (0.438–0.868)	0.006
Pathologic N stage (N1 vs. N0)	256	1.374 (0.496–3.810)	0.541
Pathologic M stage (M1 vs. M0)	500	0.855 (0.528–1.383)	0.523
Pathologic stage (Stage III&Stage IV&Stage II vs. Stage I)	529	0.620 (0.440–0.873)	0.006
Primary therapy outcome (PR&CR vs. PD&SD)	138	1.329 (0.465–3.795)	0.596
Gender (Male vs. Female)	532	0.952 (0.667–1.359)	0.785
Race (White vs. Asian&Black or African American)	525	2.091 (1.210–3.615)	0.008
Age (> 60 vs. <= 60)	532	0.655 (0.466–0.922)	0.015
Histologic grade (G3&G4 vs. G1&G2)	524	0.639 (0.452–0.902)	0.011
Serum calcium (Normal vs. Low&Elevated)	364	0.672 (0.441–1.023)	0.064
Hemoglobin (Normal vs. Low&Elevated)	452	1.201 (0.825–1.749)	0.338
Laterality (Right vs. Left)	531	1.137 (0.808–1.599)	0.462

### Analysis of genetic alterations, gene set enrichment analysis (GSEA), and interactions in ZEBs among patients with KIRC

The genetic changes of ZEBs in patients with KIRC were examined using the online tool cBioPortal. A scrutiny was conducted on three datasets, consisting of 1496 patients, to examine mutations in ZEB1 and ZEB2.The mutation rates of ZEB1 and ZEB2 were found to be 1.3% and 1%, respectively. In ZEB1, truncating mutation was the most prevalent mutation, while in ZEB2, missense mutation was the most frequently observed mutation (Supplementary Fig. 4).

**Fig. 4:**
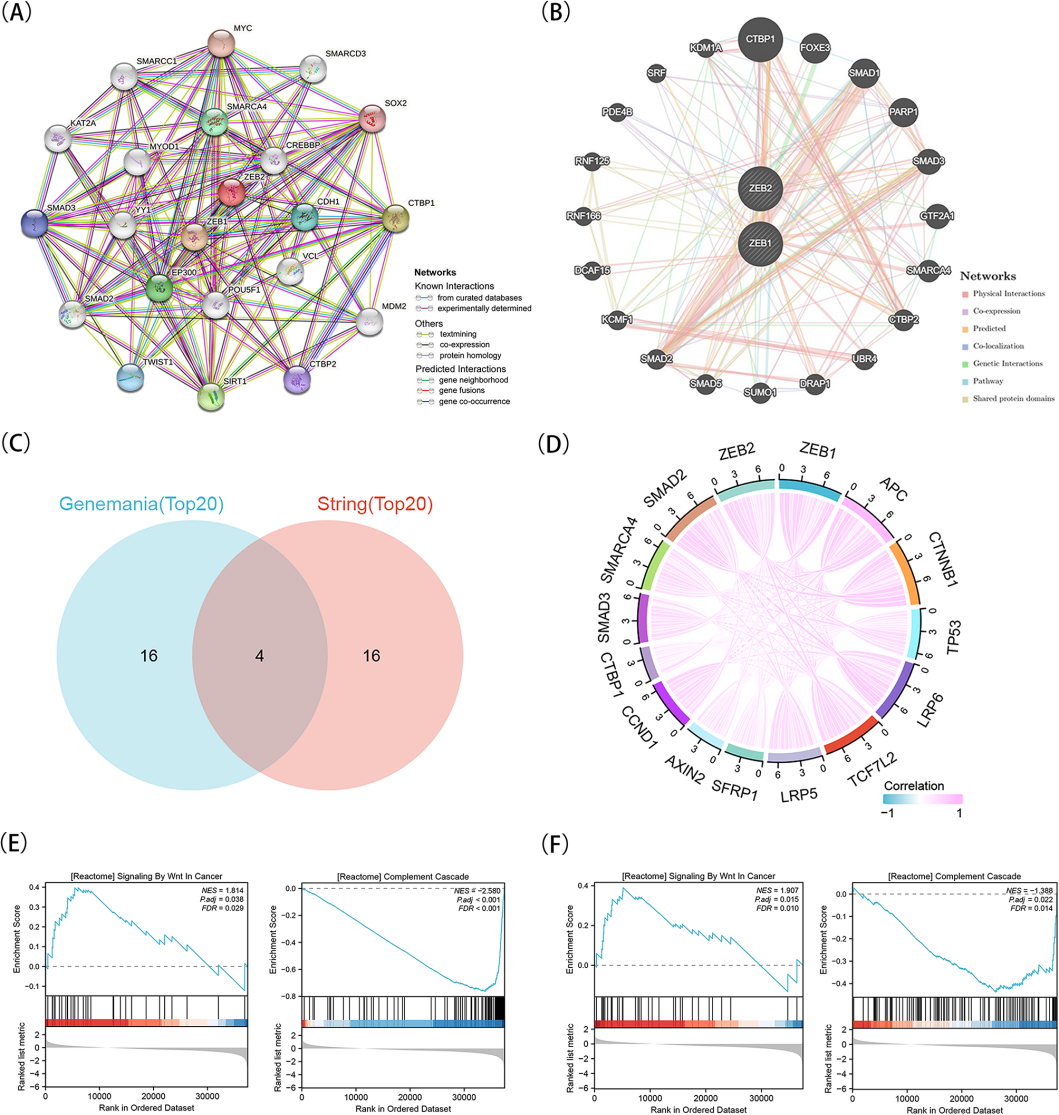
Explore the interaction genes and proteins of ZEBs, and GSEA analysis of ZEBs. A, Using GeneMania explored the gene-gene interaction network of ZEB1 and ZEB2. B, Using STRING constructed the protein-protein interaction (PPI) network of ZEB1 and ZEB2. C, the overlap of top 20 genes and proteins separately predicted by GeneMania and STRING database. D, A circos diagram revealed the wnt- pathway associated genes relation with ZEB1 and ZEB2 in KIRC. E, Reactome pathway analysis of ZEB1 using GSEA. F, Reactome pathway analysis of ZEB2 using GSEA

By using the STRING data resource, a network of protein-protein interaction (PPI) was created for ZEB1 and ZEB2. This network includes 22 nodes and 143 edges, as shown in Fig. 4A. GeneMania was utilized to build a network of gene-gene interactions involving ZEB1, ZEB2, and the neighboring altered genes. In Fig. 4B, it can be observed that there were 20 genes in the resulting network that showed significant connections with ZEB1 and ZEB2. CTBP1, SMARCA4, SMAD2, and SMAD3 were prominent genes and proteins identified in the top 20 predictions of both GeneMania and STRING, as shown by the overlap illustrated in Fig. 4C. The four genes mentioned above are linked to the Wnt signaling pathway [21–23]. Furthermore, the TCGA data resource was utilized to conduct an inquiry into the association between ZEB1 and ZEB2 and genes associated with the Wnt signaling pathway. The results showed a notable and straight connection between ZEB1 and ZEB2 with APC, CTNNB1, TP53, LRP6, TCF7L2, LRP5, SFRP1, AXIN2, CCND1, CTBP1, SMAD3, SMARCA4, and SMAD2, as illustrated in Fig. 4D. After analyzing the enrichment of ZEB1 and ZEB2 in a single gene GSEA, we have discovered two vital pathways that display notable disparities. Illustrated in Fig. 4E-F are the pathways known as Wnt Signaling in Cancer and the Complement Cascade. Moreover, we have discovered a significant association between anoikis and immune infiltration with these pathways.

### Perform GO and KEGG enrichment analysis on ZEB1 and ZEB2, as well as their co-expressed genes within the TCGA-KIRC cohort

**Fig. 5:**
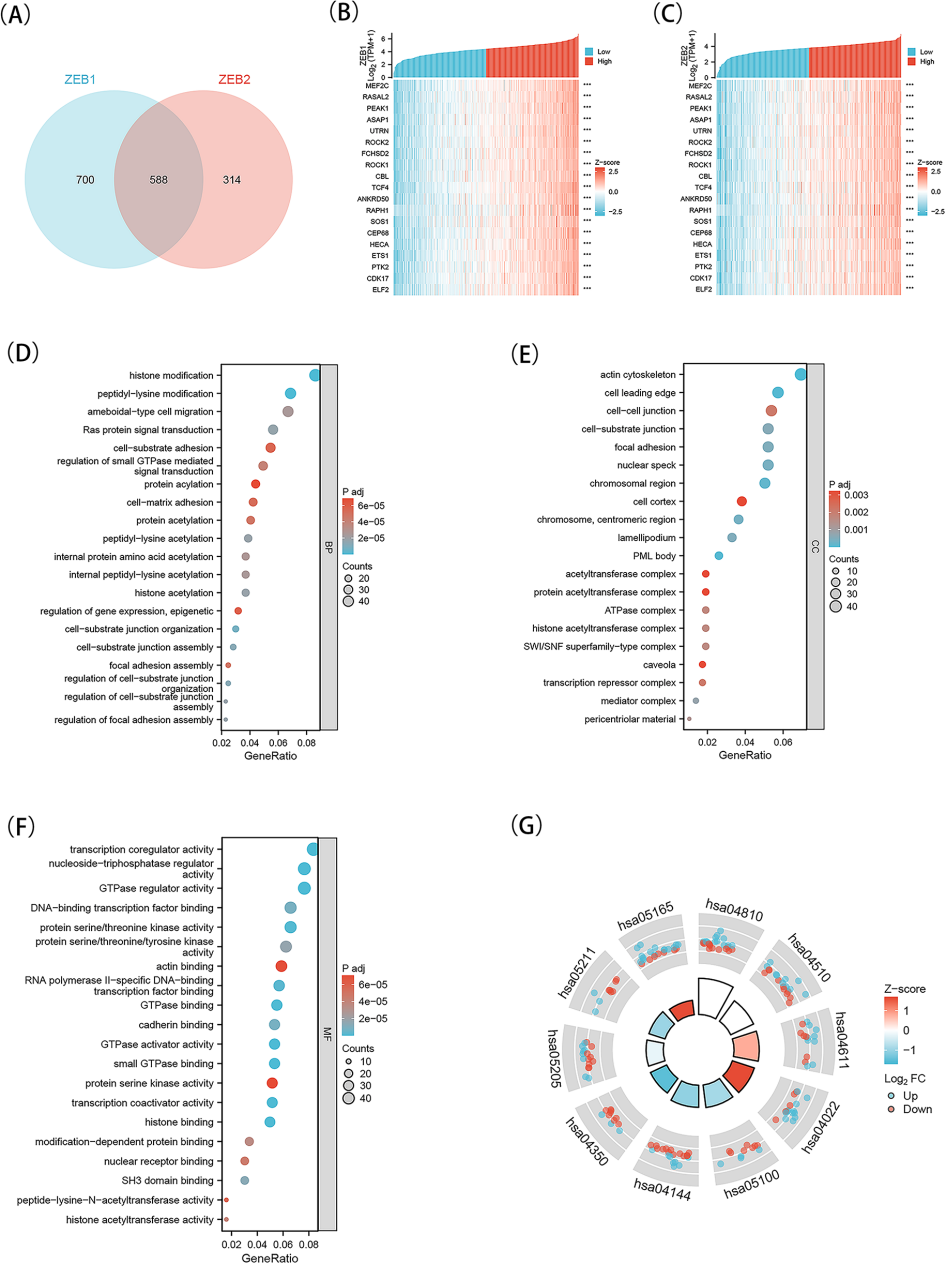
KEGG and GO enrichment analysis for ZEB1 and ZEB2 in TCGA database about KIRC. A, Intersection co-expression genes of ZEB1 and ZEB2. B, the top 20 co-expression genes positively correlated with ZEB1 in KIRC were shown by heat map. C, the top 20 co-expression genes positively correlated with ZEB2. D–G, Top 20 enrichment terms about ZEB1 and ZEB2 in BP (D), CC (E), and MF (F) categories in KIRC. G, A chord diagram showed KEGG enrichment in KIRC. Note: GO: Gene Ontology; KEGG: Kyoto Encyclopedia of Genes and Genomes; TCGA, The Cancer Genome Atlas; KIRC, kidney renal clear cell carcinoma; BP, biological process; CC, cellular component; MF, molecular function

Initially, a Venn diagram was utilized to display the 588 intersections between genes that co-express ZEB1 and ZEB2 from the TCGA transcriptome data (Fig. 5A). Subsequently, the top 20 genes that are most closely associated with ZEB1 in KIRC were selected and presented in Fig. 5B, while Fig. 5C displayed the top 20 genes that are most closely associated with ZEB2. Further analysis was conducted through KEGG and GO enrichment analyses of the 588 genes that co-expressed with ZEB1 and ZEB2. The results of the top 20 vital terms of BP, MF, and CC enrichment were presented in Fig. 5D-F. Finally, the KEGG analysis of ZEB1 and ZEB2 was presented in Fig. 5G. As previously indicated, the enrichment analysis revealed that ZEB1 and ZEB2 were significantly associated with BP related to anoikis, specifically in the regulation of cell-substrate junction organization, cell-substrate junction assembly, Ras protein signal transduction, regulation of focal adhesion assembly, ameboidal-type cell migration, cell-matrix adhesion, cell-substrate adhesion, and focal adhesion assembly. Additionally, the KEGG analysis demonstrated that ZEB1 and ZEB2 were enriched for anoikis-related processes, including Regulation of actin cytoskeleton, Focal adhesion, and Proteoglycans in cancer.

### The association between ZEB1 and ZEB2 with genes related to anoikis in KIRC

**Fig. 6:**
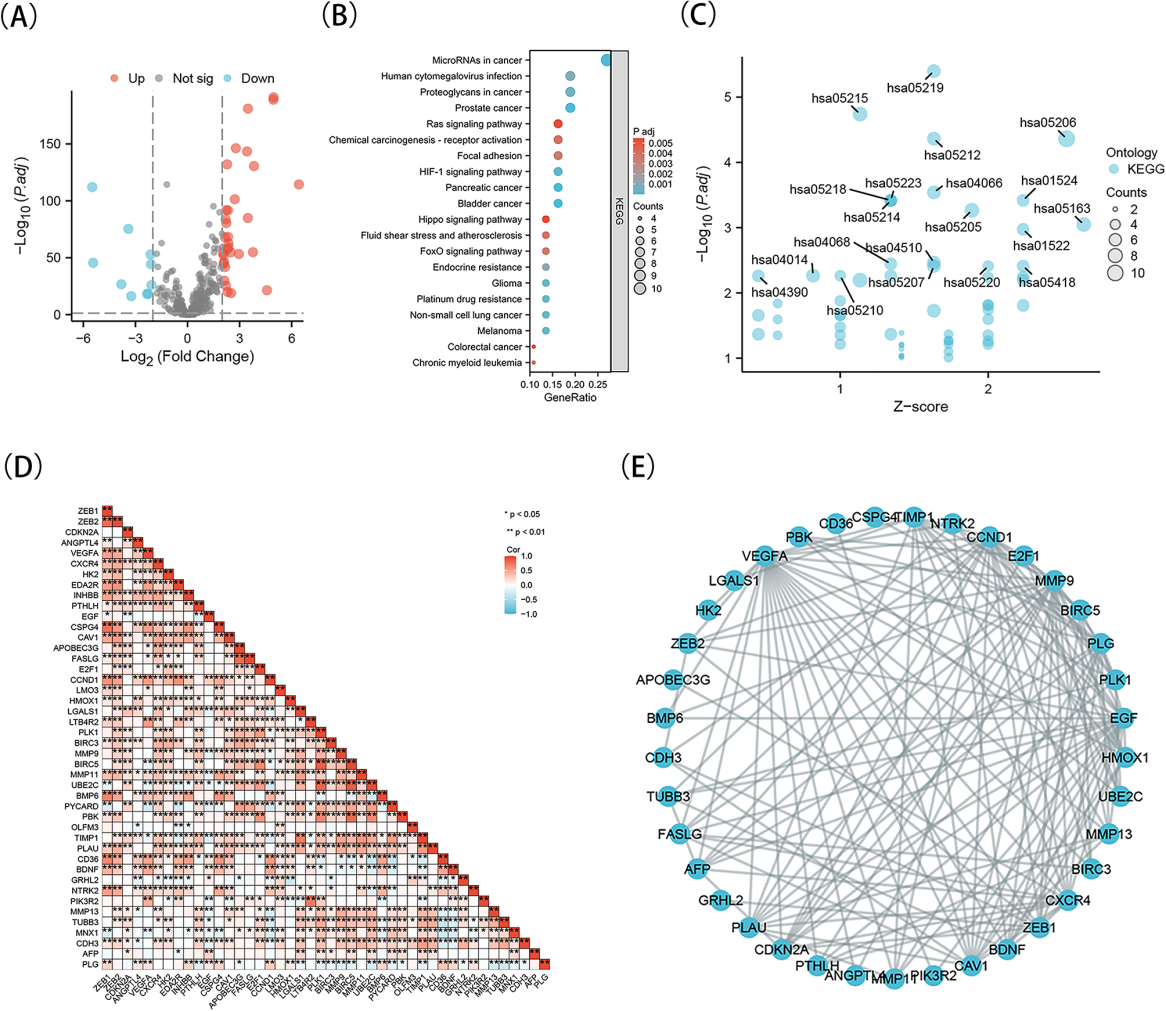
Correlation analysis of ZEB1, ZEB2 expression with anoikis-related genes in TCGA-KIRC database. A, Volcano plot presented the different expressions of anoikis-related genes and normal control genes. B, A bubble chart shows top 20 KEGG enrichment pathways for ZEB1, ZEB2 and the 42 genes in KIRC. C, A bubble chart shows top 20 KEGG enrichment pathways with z-score for ZEB1, ZEB2 and the 42 genes. D, The correlations of ZEB1, ZEB2 and the 42 genes. E, The PPI network of ZEB1, ZEB2 and the differently expressed 42 genes. *P < 0.05; **P < 0.01

In previous studies, a total of 338 genes associated with anoikis were acquired from the GeneCards database (https//www.genecards.org/). Only genes with a relevance score higher than 1.0 were included. Through integration with the TCGA-KIRC database, 42 genes linked to anoikis were found to exhibit significant differences compared to the normal controls. These differences were determined based on a P-value below 0.05 and an absolute logarithmic (base 2) fold-change value surpassing 2. Out of these genes, 32 showed upregulation, whereas 10 exhibited downregulation (Fig. 6A). According to Fig. 6D, ZEB1 was linked to 28 genes while ZEB2 was associated with 31 genes. Afterwards, a protein-protein interaction (PPI) study was performed on the 42 genes associated with anoikis. The results indicated that ZEB1 and ZEB2 exhibited associations with CCND1, BIRC5, MMP9, GRHL2, EGF, VEGFA, CDH3, CDKN2A, and CXCR4 (Fig. 6E).

The potential biological functions of 42 genes, ZEB1 and ZEB2 were investigated through the utilization of the XIANTAO platform. GO enrichment analyses and KEGG enrichment analyses were performed. The results showed that the important GO terms were connected to processes related to inhibiting cadherin-mediated cell-cell adhesion (biological processes), as well as platelet alpha granule, collagen-containing extracellular matrix, and membrane microdomain (cellular component). Furthermore, the signaling receptor activator ac tivity’s molecular function was identified (Supplementary Fig. 5). In Fig. 6B-C, the KEGG enrichment analysis indicated that Proteoglycans in cancer, Hippo signaling pathway, FoxO signaling pathway, Focal adhesion and microRNAs were associated with ZEB1, ZEB2, and 42 genes. The results indicate a potential correlation between ZEB1 and ZEB2 and anoikis in KIRC.

### Prediction of microRNAs targeting ZEB1 or ZEB2

**Fig. 7:**
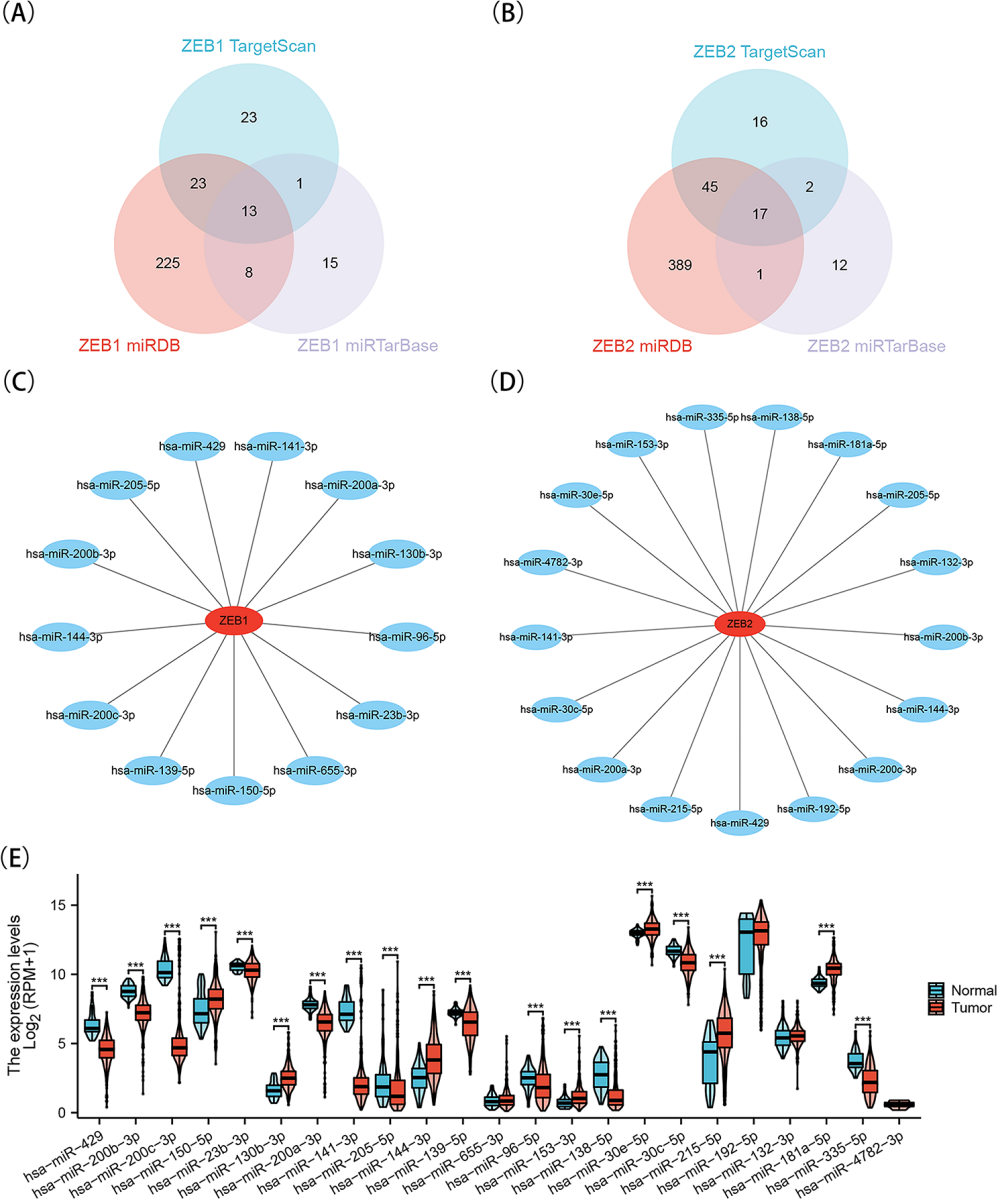
Prediction of microRNAs targeting ZEB1 or ZEB2. A, B, Venn results of microRNAs which could bind with ZEB1 (A) and ZEB2 (B) predicted by TargetScan, miRTarBase and miRDB. C, D, Network of microRNAs targeting ZEB1(C) and ZEB2(D). E, The expressions of microRNAs targeting ZEB1 or ZEB2 were shown. ***P < 0.001

Following this, by employing the overlap of three separate microRNA databases, a sum of 13 microRNAs were anticipated to aim at ZEB1, whereas 17 microRNAs were projected to target ZEB2, as illustrated in Fig. 7A-B. Figure 7C and D depict the correlation between ZEB1/2 and microRNA. Furthermore, a boxplot examination demonstrated a notable decrease in seven microRNAs that target ZEBs among KIRC patients, while 12 microRNAs targeting ZEBs showed a significant increase. Notably, four microRNAs exhibited no discernible impact (Fig. 7E). Supplemental Table 3 shows P-values and confidence intervals for microRNAs targeting ZEB1/2. The study findings suggest that ZEB1 showed a positive association with hsa-miR-139-5p and hsa-miR-23b-3p, while displaying a negative association with hsa-miR-141-3p, hsa-miR-205-5p, hsa-miR-96-5p, hsa-miR-130b-3p, hsa-miR-200b-3p, hsa-miR-200a-3p, hsa-miR-200c-3p, and hsa-miR-429 (Supplementary Fig. 6A). Furthermore, ZEB2 exhibited a positive association with hsa-miR-153-3p, hsa-miR-181a-5p, and hsa-miR-335-5p, while demonstrating a negative association with hsa-miR-429, hsa-miR-215-5p, hsa-miR-205-5p, hsa-miR-200b-3p, hsa-miR-200a-3p, hsa-miR-30e-5p, and hsa-miR-30c-5p (Supplementary Fig. 6B).

**Developing a predictive model for assessing risk in the TCGA cohort by utilizing microRNAs that target either ZEB1 or ZEB2**.

**Fig. 8:**
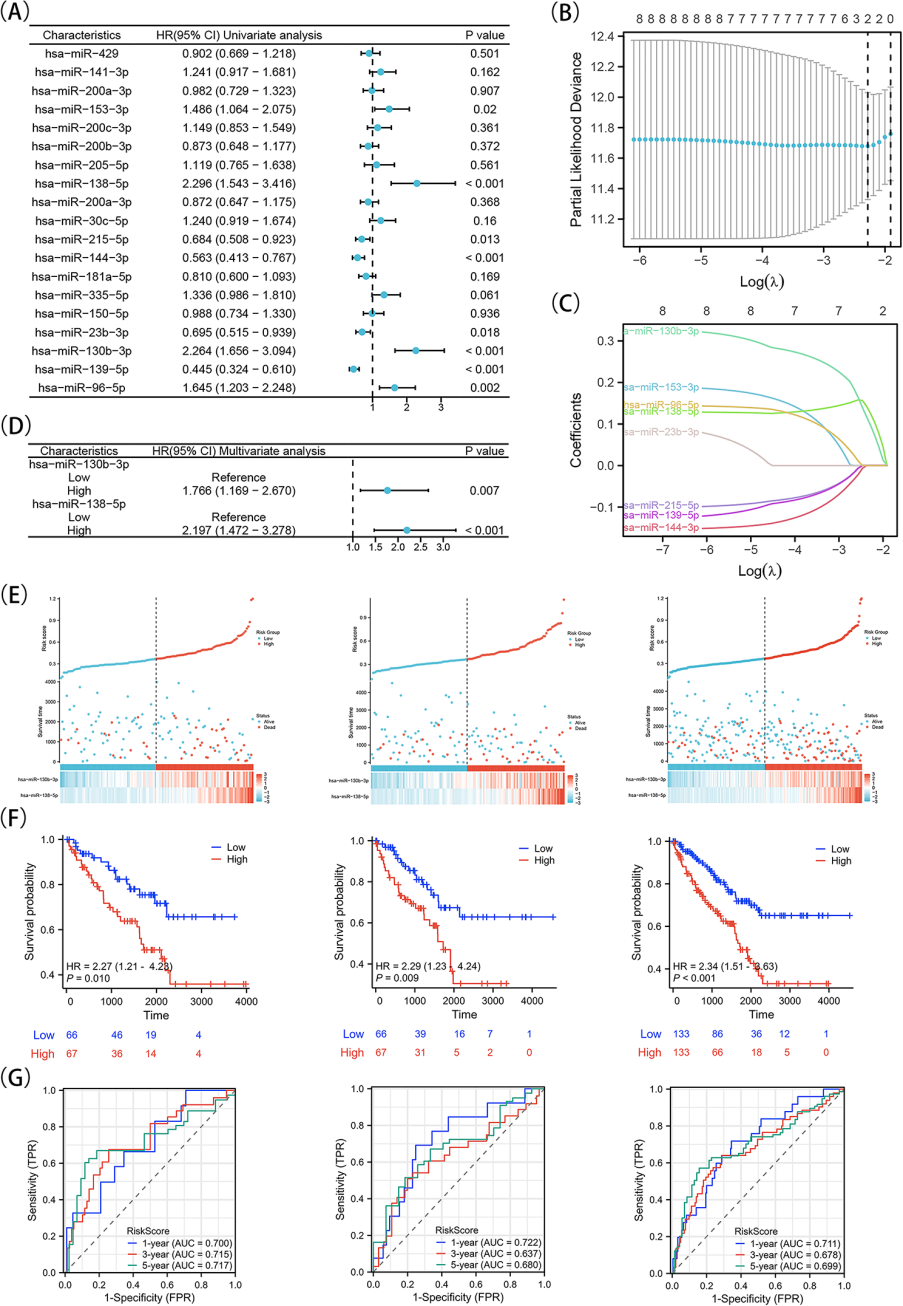
Construction and validation of a risk prognostic model based on differential microRNAs targeting ZEB1 or ZEB2 in testing, training, and entire sets. A, Univariate Cox regression analysis was performed for all differential microRNAs targeting ZEB1 or ZEB2. A value of *P* < 0.05 was considerated statistically signifcant. B, LASSO regression of the 8 OS-related microRNAs. C, Cross-validation for tuning the parameter selection in the LASSO regression. D, Multivariate Cox regression analysis was performed on the genes derived from the LASSO regression analysis. E-G, The distribution of overall survival risk scores, survival status, heatmaps of microRNA expression (E), Kaplan-Meier curves for survival status and survival time in the training, validation, and overall groups(F), time-dependent ROC curves are used to show whether microRNA signatures are predictive of 1-, 3-, and 5-year overall survival (OS) in the training, validation, and overall groups(G)

By univariate Cox regression analysis, Fig. 8A demonstrates the identification of eight microRNAs that target ZEB1 or ZEB2 with a significance level of *P* < 0.05. Out of these, four microRNAs (hsa-miR-153-3p, hsa-miR-130b-3p, hsa-miR-138-5p, and hsa-miR-96-5p) were recognized as potential hazards, whereas the other four (hsa-miR-144-3p, hsa-miR-139-5p, hsa-miR-215-5p, and hsa-miR-23b-3p) were identified as potential safeguards. Following the outcomes of the univariate Cox regression, we preformed LASSO regression analysis (Fig. 8B, C). Ultimately, a predictive model concerning microRNAs was built utilizing hsa-miR-138-5p and hsa-miR-130b-3p via LASSO regression. Following this, a regression analysis using multiple variables was performed, which indicated that hsa-miR-130b-3p and hsa-miR-138-5p were recognized as genes with potential risk (Fig. 8D). A predictive indicator was subsequently created for every cancer sample, using the subsequent equation: Risk score = (hsa-miR-130b-3p expression level × 0.096293506) + (hsa-miR-138-5p expression level × 0.124749346). To verify the prognostic ability of the microRNAs model for KIRC patients, 266 individuals were separated into two groups, the low-risk group (*n* = 133) and the high-risk group (*n* = 133), by using the median risk score threshold. In comparison to the low-risk group, the high-risk group displayed a higher fatality rate and shorter lifespan. Higher scores were a sign of a worse outlook for patients diagnosed with KIRC. Figure 8E depicts the distribution of risk scores and highlights the elevated mortality rate observed in the higher risk group when compared to the lower risk group. The heatmap analysis reveals that hsa-miR-138-5p and hsa-miR-130b-3p exhibit higher expression levels in individuals classified as high risk, as opposed to those classified as low risk. Furthermore, Kaplan-Meier analysis demonstrates that patients categorized as low risk exhibit a longer overall survival compared to those classified as high risk (Fig. 8F). Finally, the sensitivity and specificity of the 2-microRNA signature model were assessed using a receiver operating characteristic (ROC) curve. The ROC curves (AUCs) for the model at one, three, and five years were found to be 0.700, 0.715, and 0.717, respectively, indicating a favorable predictive ability (Fig. 8G, left panel). Additionally, the testing group consisting of 133 patients (Fig. 8G, middle panel) and the entire group of 266 patients were subjected to the risk score ROC analysis (Fig. 8G, right panel). Based on the aforementioned analysis, it can be concluded that this risk prognosis model is both feasible and dependable. In addition, supplementary Tables 4 and 5 describe the relationship between the expression of hsa-miR-130b-3p and hsa-miR-138-5p in KIRC with clinical characteristics. hsa-miR-130b-3p and hsa-miR-138-5p are closely related to the progression of KIRC.

### Correlation between ZEB1, ZEB2 with immunocyte infiltration

To obtain additional understanding regarding the correlation between the manifestation of ZEB1, ZEB2 and infiltrating immune cells was carried out. The investigation involved six different categories of immune cells, which consisted of CD4 + T cells, CD8 + T cells, B cells, neutrophils, dendritic cells, and macrophages. In Supplementary Fig. 7A-B, the findings suggest a strong correlation between ZEB1 and the CD4 + T cells, CD8 + T cells, neutrophils, dendritic cells, macrophages, and with little impact on B cells. In contrast, ZEB2 showed a noteworthy association with each of the six categories of immune cells. We calculated the immune scores, stromal scores, and ESTIMATE scores for every KIRC patient using the ESTIMATE method. By conducting a Spearman correlation analysis, we discovered a positive correlation between the expression of ZEB1 and both stromal scores and ESTIMATE scores. However, there was no correlation observed with the immune score (Supplementary Fig. 7C). Moreover, the expression of ZEB2 showed a clear association with the immune score, stromal score, and ESTIMATE score in KIRC (Supplementary Fig. 7D). The correlation between the manifestation of ZEB1 and ZEB2 and widely recognized T-cell checkpoints was confirmed in the TISIDB database. Significantly, in KIRC, the levels of KDR, ADORA2A, PDCD1LG2, IL10RB, and PDCD1 showed a robust association with ZEB1 expression. Furthermore, ZEB2 exhibited a notable correlation with PDCD1LG2, IL10, KDR, CSF1R, and BTLA, as indicated by the supporting data in Supplementary Fig. 7E-F.

**Fig. 9:**
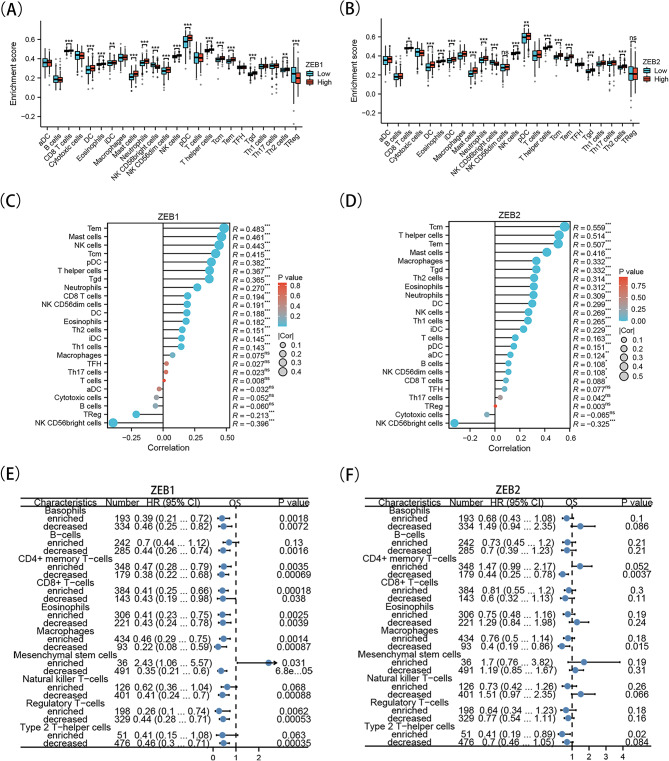
The correlations of ZEB1, ZEB2 and immune infiltration in KIRC tissues by using TCGA dataset. A, B, Differences in immune cell infiltration in ZEB1 (A) and ZEB2 (B) high and low expression groups through ssGSEA. C, D, lollipop charts showed the correlation of ZEB1 (C) and ZEB2 (D) with all 24 types of immune cells. E, F, Forest plots show the prognostic value of ZEB1 (E) and ZEB2 (F) expression according to different immune cell subgroups in KIRC patients

Results for the analysis of immune cell infiltration for ZEB1 and ZEB2 are shown in Fig. 9A-B, respectively. Infiltration of CD8 T cells, DC, eosinophils, iDC, mast cells, neutrophils, NK CD 56bright cells, NK CD56dim cells, NK cells, pDC, T helper cells, Tcm, Tem, Tgd, Th2 cells, and TReg varied between the high and low ZEB1 groups (Fig. 9A). In the same way, the groups with high-ZEB2 and low-ZEB2 showed varying levels of infiltration by the mentioned types of immune cells (Fig. 9B).

The primary focus of Fig. 9C is to showcase the presence of ZEB1 in the tumor microenvironment (TME) of KIRC. ZEB1 showed a favorable association with different types of immune cells, such as Tem cells, mast cells, NK cells, T helper cells, Tcm cells, pDCs, Tgd cells, neutrophils, DCs, Eosinophils, NK CD56dim cells, CD8 T cells, Th1 cells, iDCs, and Th2 cells. According to the research, ZEB1 showed an inverse relationship with NK CD56 (bright) cells and regulatory T cells (TReg), whereas ZEB2 displayed a direct correlation with T helper cells, Tcm, Tem, mast cells, eosinophils, macrophages, Tgd, DC, neutrophils, NK cells, Th2 cells, Th1 cells, iDC, T cells, pDC, aDC, B cells, NK CD56dim cells, and CD8 T cells. Furthermore, ZEB2 demonstrated an inverse association with NK CD56bright cells, as depicted in Fig. 9D.

This study was to explore the possible influence of immune infiltration on the prognosis of KIRC by analyzing ZEB1, ZEB2 expression. The examination uncovered the correlation between ZEB1, ZEB2 and unfavorable prognoses in KIRC. In order to explore the expression of ZEB1, ZEB2 effect on the prognosis of KIRC, we examined their levels in the respective subcategories of immune cells. The results shown in Fig. 9E suggest that KIRC patients with elevated ZEB1 expression demonstrate limited infiltration of type 2 T-helper cells, natural killer T-cells and B-cell. Additionally, a noteworthy correlation was found between ZEB1 levels and prognosis of KIRC in a cohort consisting of different levels of regulatory CD8 + T cells, CD4 + memory-T cells, T cells, basophils, eosinophils, macrophages, and Mesenchymal stem cells (Fig. 9E). Significantly, KIRC individuals showing increased ZEB2 levels and limited infiltration of CD4 + memory-T cells and macrophages demonstrated a worse prognosis. Furthermore, a strong association was found between increased levels of Type 2 T-helper cells and elevated ZEB2 levels, which significantly impacted the prognosis of KIRC (Fig. 9F). The results indicate that the invasion of ZEB1 and ZEB2 into the immune system may have a potential effect on the prognosis of individuals diagnosed with KIRC. Furthermore, supplementary Table 6 presents the correlation between the expression levels of hsa-miR-130b-3p and hsa-miR-138-5p and immune evasion genes in KIRC. The findings suggest a potential association between hsa-miR-130b-3p and hsa-miR-138-5p and immune evasion mechanisms.

**Fig. 10:**
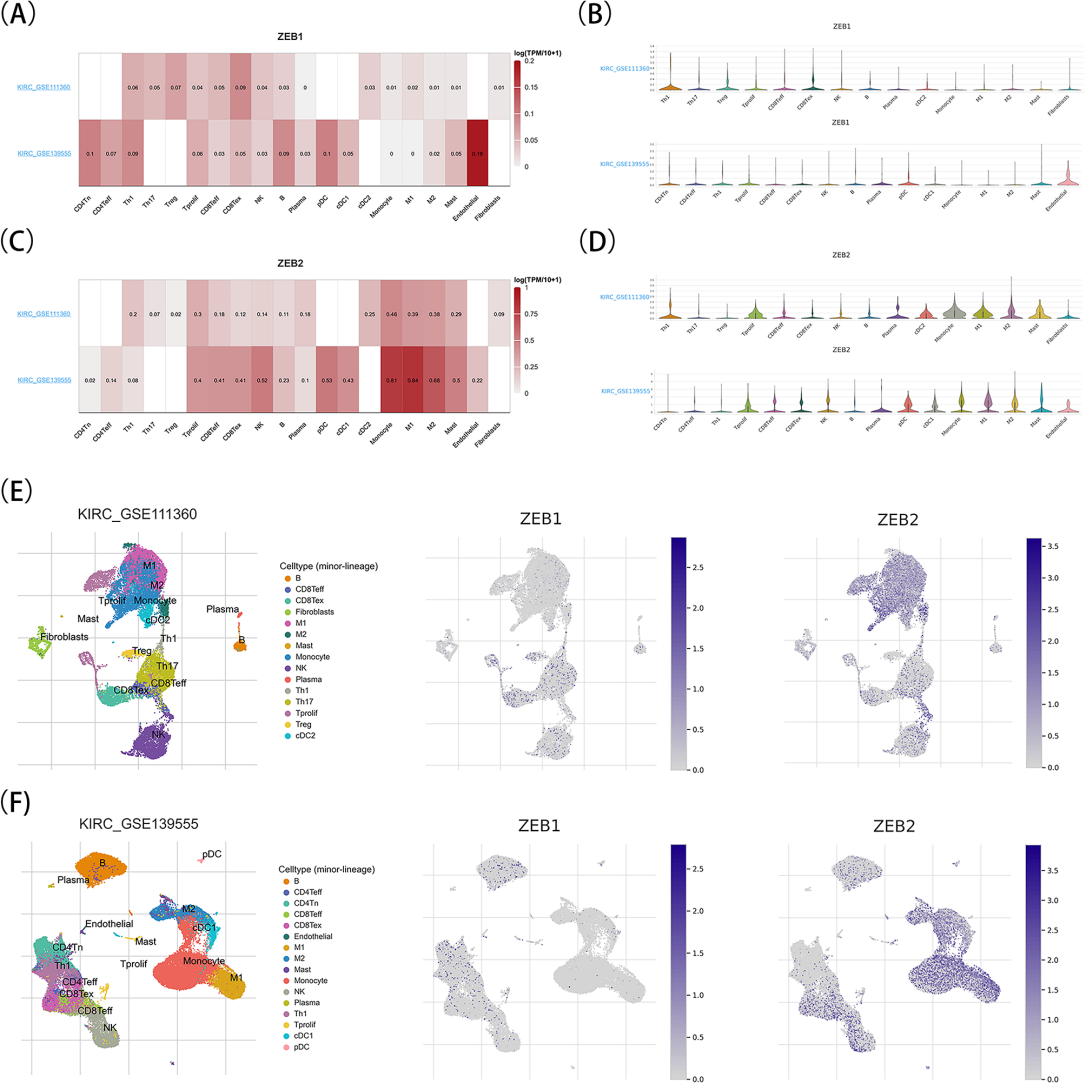
Expression of ZEB1 and ZEB2 in scRNA-seq landscapes by TISCH. A, B, Heatmaps of ZEB1 (A) and ZEB2 (B) expression displayed heterogeneity in different clusters of cells in KIRC_GSE111360 and KIRC_GSE139555 datasets. C, D, Violin diagrams depict the ZEB1 (C) and ZEB2. (D) expression in different immune cells across each dataset analyzed. E, F, Expression of ZEB1 (E) and ZEB2 (F) in GSE111360 and in GSE139555 datasets after Uniform Manifold Approximation and Projection (UMAP) processing

In order to improve the thoroughness and strength of the results, analysis of two separate scRNA-seq datasets indicated ZEB1 expression was principally detected in endothelial cells. Additionally, CD4 naive T cells (CD4Tn), B cells (B), natural killer cells (NK), Type 1 T helper cells (Th1), CD8 + T cells (CD8Tex), CD8 effector T cells (CD4Teff), and natural killer cells (NK) exhibited reduced levels of ZEB1 expression (Fig. 10A, B, E and F). Furthermore, ZEB2 exhibited prominent expression in diverse immune cell populations, such as M1 and M2 macrophages, monocytes, fatigued pDCs, NK cells, Mast cells, cDC1, CD8Tex, CD8 Teff, Tprolif, B cells, and endothelial cells (Fig. 10C, D, E, and F). The possible importance of ZEB1 and ZEB2 in the TME of KIRC expands to their influence on both stromal and immune cells.

#### Functional analysis of ZEBs with CancerSEA

**Fig. 11:**
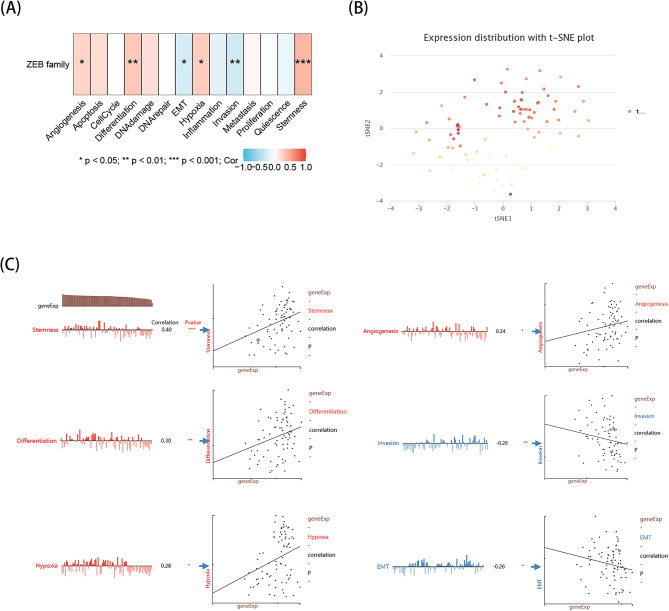
The expression levels of ZEB1 and ZEB2 at single-cell levels in RCC. A, The relationship between ZEBs expression and different functional states in RCC was explored by the CancerSEA tool. B, ZEBs expression profiles were shown at single cells from RCC by T-SNE diagram. C, Detailed functional correlations in RCC. *p < 0.05; **p < 0.01; ***p < 0.001

The application of single-cell sequencing technology offers an unmatched opportunity to accurately decipher the functional conditions of cancer cells at an individual cellular level. To determine the expression of the ZEB family in RCC, we analyzed the functional states of the ZEB family in RCC by examining published gene profiling studies available on CancerSEA (http //biocc.hrbmu.edu.cn/CancerSEA/). The analysis uncovered a strong association between ZEB1 and ZEB2 with RCC. In renal cell carcinoma (RCC), the expression of ZEBs shows a positive correlation with four functional conditions: angiogenesis, hypoxia, differentiation, and stemness. This correlation is supported by Spearman’s coefficients of 0.24, 0.28, 0.30, and 0.40, respectively, and a significance level below 0.05 (*P* < 0.05). In contrast, ZEBs expression shows a negative correlation with EMT and invasion. The Spearman’s coefficients for both are − 0.26 and − 0.29, respectively, with a significance level of *P* < 0.05, as illustrated in Fig. 11A, C. Using t-SNE, the analysis was conducted on the distribution of ZEBs expression in renal carcinoma cells, which unveiled that cells demonstrating elevated ZEBs expression had a propensity to form clusters. The aforementioned observation suggests that this distribution pattern could potentially aid in the malignant advancement of RCC, as illustrated in Fig. 11B.

## Discussion

It was found that RCC is the second-most frequently occurring urological malignancy, and kidney renal clear cell carcinoma (KIRC) is most prevalent [24]. The clinical management of RCC involves the use of different treatment approaches, such as targeted therapy, chemotherapy, radiofrequency ablation, surgical procedures, and immunotherapy [25]. The uncertainty surrounding the predictive significance of a signature or indicator for ccRCC patients persists. Although medical professionals have made significant progress in treating ccRCC, the prognosis for individuals with advanced and metastatic conditions remains unsatisfactory [26]. Over a span of three years, the approximate survival rate for individuals with lymph node metastases is around 20–30%. Patients with advanced RCC often receive targeted therapies, like sunitinib, but resistance to these treatments usually emerges within 6–15 months, leading to an overall unsatisfactory survival rate [27–29]. Hence, it is crucial to pursuit possible mechanism that contribute to RCC and to establish reliable indicators for prognosis.

By conducting bioinformatics analyses on publicly accessible data sources like TIMER2.0, UALCAN, and TCGA, we have noticed increased expressions of ZEB1 and ZEB2 in KIRC when compared to normal kidneys. Previous reports have indicated that ZEB1 and ZEB2 might function as oncogenes, contributing to the development of ccRCC [30, 31]. Furthermore, our inquiry uncovered that the undermethylation of ZEB1 and ZEB2 might contribute to their increased expression in KIRC tumors. Following this, a study was performed on KIRC patients to determine the clinical prognostic importance of ZEB1 and ZEB2.Significantly, in patients with KIRC, increased levels of ZEB1 and ZEB2 showed notable correlations with gender, histological grade, age, TNM stage, and clinical stage. Moreover, the Kaplan-Meier analysis revealed a considerably higher rate of survival among KIRC individuals exhibiting elevated ZEB1 or ZEB2 levels in contrast to those displaying lower expression. The results indicate that ZEB1 and ZEB2 could potentially function as prognostic indicators in KIRC and promote the progress of precision oncology targeting.

In both GO and KEGG, the TCGA-KIRC cohort demonstrates a concentration of co-expressed genes related to ZEB1 and ZEB2.According to the GO analysis, ZEB1 and ZEB2 are involved in multiple biological processes, such as Ras protein signaling, controlling the assembly of focal adhesion, ameboidal-type cell migration, actin cytoskeleton, focal adhesion, and binding to cadherin. Anoikis has also been associated with these processes in previous studies [32]. In the context of integrin-mediated adhesion, the activation of focal adhesion kinase (FAK) has been observed to inhibit anoikis, indicating the crucial role of focal adhesion in this process [33, 34]. Moreover, a GSEA investigation has revealed a noteworthy correlation between ZEB1 and ZEB2 with Signaling by Wnt in Cancer and Complement Cascade pathways, which are strongly connected to both anoikis and immune infiltration [35, 36].

The mechanism of anoikis defense aims to hinder the abnormal growth of shed cells and stop their improper attachment to a matrix [37]. The occurrence of anoikis is strongly linked to the development of tumors and the ability to resist treatment [38]. The manifestation of anoikis is highly associated with tumor formation and resistance to treatment [39]. As tumors disseminate, cancerous cells migrate from their primary site and acclimate to anomalous growth in distant locations. Numerous techniques have been exhibited to attain resistance to anoikis in tumors, such as the regulation of the expression of cell adhesion molecules and the management of oxidative stress [40]. However, additional research is necessary to determine the exact process through which KIRC evade anoikis. Previous research has suggested that ccRCC cells resistant to anoikis exhibit uncontrolled growth and a lack of responsiveness to detachment-induced apoptosis. In addition, the studied population exhibited an increase of Tyrosine receptor kinase B (TrkB) compared to the parental ccRCCs [41]. According to a recent study, the use of quinazoline-based medications to activate focal adhesion survival signaling has been found to trigger anoikis in RCC. This discovery presents a hopeful treatment strategy for addressing this illness [42].

Genes linked to anoikis include ZEB1 and ZEB2. In particular, the ZEB1 gene has been discovered to contribute to the development of resistance to anoikis, while also facilitating migration and invasion in reaction to TrkB37 [43]. Furthermore, studies have indicated that excessive expression of RARA results in an elevation of ZEB2, a crucial element in promoting epithelial-mesenchymal transition (EMT) and facilitating the evasion of anoikis and proliferation of breast cancer cells [44]. Anoikis resistance and oncogenic EMT are closely related. Breakdown of E-cadherin expression or function is a characteristic of EMT [45–47]. Anoikis can be avoided in certain contexts by targeting the E-cadherin gene [48]. For example, a mouse mammary tumor model or a mammary epithelial cell line expressing E-cadherin knockout confer anoikis resistance [49]. This finding suggests that EMT-promoting transcription factors such as ZEB1 and ZEB2 may potentially block anoikis by directly regulating apoptosis control genes and suppressing the expression of E-cadherin. ZEB1 and ZEB2, in relation to KIRC, have been discovered to have a strong association with 42 distinct genes and proteins that play a role in anoikis. Moreover, the examination of KEGG enrichment has indicated that the process of MicroRNAs in cancer is predominantly linked to ZEB1, ZEB2, and the 42 genes related to anoikis. The imbalance of miRNAs plays a major role in cancer cell survival strategies, such as resistance to anoikis, which is essential for facilitating metastasis [35]. An example of this is when microRNA-6744-5p induces anoikis in breast cancer by specifically targeting the NAT1 enzyme [50]. On the other hand, diminished levels of miR-30a in metastatic HCC promote Beclin 1 and Atg5-mediated autophagy, leading to increased resistance against anoikis [9]. The results of our study suggest that the prognostic value for KIRC patients can be determined by the signatures of hsa-miR-130b-3p and hsa-miR-138-5p. Previous studies have shown that exosome miR-130b-3p, which specifically targets SIK1, has the ability to hinder the advancement of medulloblastoma [51]. Furthermore, early lupus nephritis patients with kidney damage have shown increased levels of serum miR-130b-3p [52]. In the case of endometrial cancer, the increase in miR-130b levels has been demonstrated to decrease the expression of ZEB1 and weaken the process of EMT [53]. Moreover, there is a notable correlation between RCC and hsa-miR-138-5p, which has a vital function in advancing cancer growth through the regulation of ZEB2 [54]. Furthermore, viruses have been associated with the regulation of apoptosis, autophagy, and anoikis pathways through the modulation of miRNAs [55]. Promoting anoikis, the miR-200 family, specifically miR-200c, directly targets ZEB1 and ZEB2 [56].Within KIRC, hsa-miR-130b-3p and hsa-miR-138-5p have been identified as inhibitors of the expression of ZEB1 and ZEB2, respectively. These microRNAs may play a role in the regulation of anoikis in KIRC by targeting ZEB1 and ZEB2.

ZEB1 and ZEB2 are expressed by myeloid and lymphoid immune cells, including dendritic cells, macrophages, monocytes, B cells, T cells, and NK cells, according to the present knowledge [57]. The dissemination of this knowledge has been extensive. ZEB1 has been discovered to promote the generation of immune-suppressive cells and chemokines within the tumor microenvironment (TME), leading to the formation of an immunosuppressive barrier around infiltrating cancer cells by activating immune checkpoints, leading to an immunosuppressive microenvironment [58, 59]. In the context of colon cancer, ZEB2 has been recognized as a crucial participant in the immune microenvironment [60]. Through GSEA enrichment analyses, the current investigation has revealed the participation of ZEB1 and ZEB2 in various pathways, specifically the complement cascade. It is worth mentioning that the upregulation of ZEB1 expression is caused by the activation of p38 mitogen-activated protein kinase (MAPK) through C5a secretion from mesenchymal stem-like cells [61]. Complement systems, being ancient and indispensable constituents of innate immunity, impede immune responses that rely on cellular proliferation [36, 62]. Furthermore, the initiation of the traditional complement pathway by MUC1 possesses the capability to regulate the immune response within the clear cell renal cell carcinoma (ccRCC) microenvironment and control immune infiltration, ultimately leading to the promotion of an immunosuppressive microenvironment [63]. It is still not clear how ZEB1/2 interacts with complement to affect KIRC immunoinfiltration. According to our study, diminished amounts of ZEB1 and ZEB2 in kidney cancer are associated with a decline in the infiltration of different immune cells, such as B cells, neutrophils, CD4 + T cells, dendritic cells, CD8 + T cells, and macrophages. The results of our study indicate that ZEB1 and ZEB2 have the potential to be effective targets for immune-related treatment in KIRC. Nevertheless, additional inquiry is required to clarify the exact way in which ZEB1 and ZEB2 engage with the tumor-immune microenvironment.

Although there has been advancement in current studies regarding the connection between ZEB1, ZEB2, anoikis, and immune infiltration in KIRC, there are still some existing constraints. Despite investigating the association between these factors in KIRC patients, the precise mechanisms through which ZEB1 and ZEB2 trigger anoikis and immune infiltration remain uncertain. Another observation indicated that the presence of ZEB1 and ZEB2 was weak among KIRC patients, and their expression was associated with immune infiltration. However, further investigation is crucial to clarify the evident regulatory processes that control the growth of tumors, their spread, and the invasion of the immune system, which involve ZEB1 and ZEB2. In current research, there have been multiple examinations conducted on the mRNA makeup of ZEB1 and ZEB2, which is the third point of analysis. However, a more comprehensive inquiry based on protein composition may enhance the persuasiveness of the findings. Furthermore, our investigation uncovered a noteworthy correlation between ZEB1 and ZEB2 with both detachment-induced cell death and immune cell infiltration. Nonetheless, the exact association between immune infiltration and anoikis, along with the possible participation of ZEB1 and ZEB2 in these interconnected mechanisms, is yet to be clarified. Furthermore, a subsequent inquiry uncovered that ZEB1 and ZEB2 have a vital function in enhancing anoikis and the invasion of immune cells in the tumor microenvironment among patients with KIRC. As a result, these findings contribute to our understanding of the characteristics of ZEB1 and ZEB2, as well as their practical implications in predicting and treating KIRC.

## Conclusion

In summary, in this systematic bioinformatics analysis of KIRC, our research has revealed that the ZEB family serves as a prognostic marker for KIRC, exhibiting a strong association with anoikis and immune infiltration, primarily under the regulation of microRNA.

### Electronic supplementary material

Below is the link to the electronic supplementary material.

Supplementary Material 1

## Data Availability

No datasets were generated or analysed during the current study.
