# Supplementary material for: ZEB family is a prognostic biomarker and correlates with anoikis and immune infiltration in kidney renal clear cell carcinoma
Source: BMC Med Genomics. 2024 Jun 5;17:153. doi: 10.1186/s12920-024-01895-7 (PMC11151722; doi:10.1186/s12920-024-01895-7)
Supplement: Supplementary file 1 — Supplementary Material 1 [file 12920_2024_1895_MOESM1_ESM.docx]

Supplementary Material

Supplementary Table 1 Relationship between ZEB1 mRNA expression and clinical characteristics in KIRC.

| Characteristics | Low expression of ZEB1 | High expression of ZEB1 | P value |
| --- | --- | --- | --- |
| n | 266 | 266 |  |
| Pathologic T stage, n (%) |  |  | < 0.001 |
| T1 | 114 (21.4%) | 158 (29.7%) |  |
| T2 | 44 (8.3%) | 25 (4.7%) |  |
| T3&T4 | 108 (20.3%) | 83 (15.6%) |  |
| Pathologic N stage, n (%) |  |  | 0.333 |
| N0 | 120 (46.9%) | 120 (46.9%) |  |
| N1 | 10 (3.9%) | 6 (2.3%) |  |
| Pathologic M stage, n (%) |  |  | 0.002 |
| M0 | 197 (39.4%) | 224 (44.8%) |  |
| M1 | 52 (10.4%) | 27 (5.4%) |  |
| Pathologic stage, n (%) |  |  | < 0.001 |
| Stage I | 111 (21%) | 155 (29.3%) |  |
| Stage II | 33 (6.2%) | 24 (4.5%) |  |
| Stage III&Stage IV | 121 (22.9%) | 85 (16.1%) |  |
| Primary therapy outcome, n (%) |  |  | 0.664 |
| PD&SD | 8 (5.8%) | 8 (5.8%) |  |
| PR&CR | 54 (39.1%) | 68 (49.3%) |  |
| Gender, n (%) |  |  | 0.084 |
| Female | 84 (15.8%) | 103 (19.4%) |  |
| Male | 182 (34.2%) | 163 (30.6%) |  |
| Race, n (%) |  |  | 0.706 |
| Asian | 4 (0.8%) | 4 (0.8%) |  |
| Black or African American | 31 (5.9%) | 25 (4.8%) |  |
| White | 228 (43.4%) | 233 (44.4%) |  |
| Age, n (%) |  |  | 0.056 |
| <= 60 | 121 (22.7%) | 143 (26.9%) |  |
| > 60 | 145 (27.3%) | 123 (23.1%) |  |
| Histologic grade, n (%) |  |  | < 0.001 |
| G1 | 4 (0.8%) | 10 (1.9%) |  |
| G2 | 89 (17%) | 139 (26.5%) |  |
| G3&G4 | 167 (31.9%) | 115 (21.9%) |  |
| Serum calcium, n (%) |  |  | 0.018 |
| Low | 94 (25.8%) | 110 (30.2%) |  |
| Normal | 90 (24.7%) | 60 (16.5%) |  |
| Elevated | 7 (1.9%) | 3 (0.8%) |  |
| Hemoglobin, n (%) |  |  | 0.859 |
| Low | 136 (30.1%) | 126 (27.9%) |  |
| Normal | 97 (21.5%) | 88 (19.5%) |  |
| Elevated | 2 (0.4%) | 3 (0.7%) |  |
| Laterality, n (%) |  |  | 0.461 |
| Left | 129 (24.3%) | 121 (22.8%) |  |
| Right | 136 (25.6%) | 145 (27.3%) |  |
| ZEB2, n (%) |  |  | < 0.001 |
| Low | 215 (40.4%) | 51 (9.6%) |  |
| High | 51 (9.6%) | 215 (40.4%) |  |

Supplementary Table 2 Relationship between ZEB1 mRNA expression and clinical characteristics in KIRC.

| Characteristics | Low expression of ZEB2 | High expression of ZEB2 | P value |
| --- | --- | --- | --- |
| n | 266 | 266 |  |
| Pathologic T stage, n (%) |  |  | 0.007 |
| T1 | 120 (22.6%) | 152 (28.6%) |  |
| T2 | 44 (8.3%) | 25 (4.7%) |  |
| T3&T4 | 102 (19.2%) | 89 (16.7%) |  |
| Pathologic N stage, n (%) |  |  | 0.540 |
| N0 | 124 (48.4%) | 116 (45.3%) |  |
| N1 | 7 (2.7%) | 9 (3.5%) |  |
| Pathologic M stage, n (%) |  |  | 0.523 |
| M0 | 202 (40.4%) | 219 (43.8%) |  |
| M1 | 41 (8.2%) | 38 (7.6%) |  |
| Pathologic stage, n (%) |  |  | 0.019 |
| Stage I | 117 (22.1%) | 149 (28.2%) |  |
| Stage II | 34 (6.4%) | 23 (4.3%) |  |
| Stage III&Stage IV | 113 (21.4%) | 93 (17.6%) |  |
| Primary therapy outcome, n (%) |  |  | 0.595 |
| PD&SD | 9 (6.5%) | 7 (5.1%) |  |
| PR&CR | 60 (43.5%) | 62 (44.9%) |  |
| Gender, n (%) |  |  | 0.785 |
| Female | 92 (17.3%) | 95 (17.9%) |  |
| Male | 174 (32.7%) | 171 (32.1%) |  |
| Race, n (%) |  |  | 0.006 |
| Asian | 3 (0.6%) | 5 (1%) |  |
| Black or African American | 39 (7.4%) | 17 (3.2%) |  |
| White | 220 (41.9%) | 241 (45.9%) |  |
| Age, n (%) |  |  | 0.015 |
| <= 60 | 118 (22.2%) | 146 (27.4%) |  |
| > 60 | 148 (27.8%) | 120 (22.6%) |  |
| Histologic grade, n (%) |  |  | 0.020 |
| G1 | 4 (0.8%) | 10 (1.9%) |  |
| G2 | 102 (19.5%) | 126 (24%) |  |
| G3&G4 | 155 (29.6%) | 127 (24.2%) |  |
| Serum calcium, n (%) |  |  | 0.156 |
| Low | 99 (27.2%) | 105 (28.8%) |  |
| Normal | 87 (23.9%) | 63 (17.3%) |  |
| Elevated | 4 (1.1%) | 6 (1.6%) |  |
| Hemoglobin, n (%) |  |  | 0.611 |
| Low | 142 (31.4%) | 120 (26.5%) |  |
| Normal | 92 (20.4%) | 93 (20.6%) |  |
| Elevated | 3 (0.7%) | 2 (0.4%) |  |
| Laterality, n (%) |  |  | 0.461 |
| Left | 129 (24.3%) | 121 (22.8%) |  |
| Right | 136 (25.6%) | 145 (27.3%) |  |
| ZEB1, n (%) |  |  | < 0.001 |
| Low | 215 (40.4%) | 51 (9.6%) |  |
| High | 51 (9.6%) | 215 (40.4%) |  |

Supplementary Table 3 p-values and confidence intervals of microRNAs targeting ZEB.

| MicroRNA | Group I | Group J | Statistic | Difference (J-I) | 95% CI | p-value | FDR |
| --- | --- | --- | --- | --- | --- | --- | --- |
| hsa-miR-153-3p | Normal | Tumor | 6910 | 0.32974 | 0.20645 - 0.46797 | 3.01E-07 | 4.73E-07 |
| hsa-miR-141-3p | Normal | Tumor | 3.62E+04 | -5.3175 | -0.4656 | 5.09E-36 | 5.60E-35 |
| hsa-miR-200a-3p | Normal | Tumor | 3.53E+04 | -1.2658 | -0.3545 | 8.27E-30 | 3.64E-29 |
| hsa-miR-429 | Normal | Tumor | 3.60E+04 | -1.6953 | -0.4434 | 2.41E-32 | 1.33E-31 |
| hsa-miR-200c-3p | Normal | Tumor | 3.71E+04 | -5.5414 | -0.4372 | 1.76E-36 | 3.87E-35 |
| hsa-miR-200b-3p | Normal | Tumor | 3.62E+04 | -1.5521 | -0.3418 | 9.26E-33 | 6.79E-32 |
| hsa-miR-205-5p | Normal | Tumor | 1.41E+04 | -0.51372 | -0.56722 | 0.0007 | 0.000810526 |
| hsa-miR-138-5p | Normal | Tumor | 2.03E+04 | -1.5044 | -0.5625 | 2.47E-20 | 6.79E-20 |
| hsa-miR-30e-5p | Normal | Tumor | 1.29E+04 | 0.29681 | 0.16812 - 0.42442 | 5.56E-06 | 7.65E-06 |
| hsa-miR-30c-5p | Normal | Tumor | 3.20E+04 | -0.85922 | -0.33209 | 2.42E-19 | 5.92E-19 |
| hsa-miR-215-5p | Normal | Tumor | 8827 | 1.7606 | 1.3042 - 2.2327 | 1.06E-13 | 1.94E-13 |
| hsa-miR-144-3p | Normal | Tumor | 8296 | 1.388 | 1.0585 - 1.723 | 1.61E-14 | 3.22E-14 |
| hsa-miR-181a-5p | Normal | Tumor | 4903 | 1.0114 | 0.87667 - 1.1428 | 1.31E-24 | 4.80E-24 |
| hsa-miR-335-5p | Normal | Tumor | 3.25E+04 | -1.4954 | -0.4784 | 1.64E-23 | 5.15E-23 |
| hsa-miR-150-5p | Normal | Tumor | 1.27E+04 | 0.84402 | 0.52942 - 1.14 | 2.04E-06 | 2.99E-06 |
| hsa-miR-23b-3p | Normal | Tumor | 2.46E+04 | -0.25515 | -0.28057 | 0.0002 | 0.000244444 |
| hsa-miR-130b-3p | Normal | Tumor | 6934 | 0.87486 | 0.7001 - 1.049 | 1.78E-18 | 3.92E-18 |
| hsa-miR-139-5p | Normal | Tumor | 2.84E+04 | -0.72442 | -0.46377 | 1.28E-10 | 2.17E-10 |
| hsa-miR-96-5p | Normal | Tumor | 2.40E+04 | -0.62967 | -0.51664 | 8.92E-06 | 1.15E-05 |
| hsa-miR-655-3p | Normal | Tumor | 8843 | 0.042827 | -0.259648 | 0.5208 | 0.5208 |
| hsa-miR-192-5p | Normal | Tumor | 1.83E+04 | 0.13106 | -0.75381 | 0.4748 | 0.497409524 |
| hsa-miR-132-3p | Normal | Tumor | 1.73E+04 | 0.1295 | -0.354443 | 0.154 | 0.1694 |

Supplementary Table 4 Relationship between hsa-miR-130b-3p expression and clinical characteristics in KIRC.

| Characteristics | Low expression of hsa-miR-130b-3p | High expression of hsa-miR-130b-3p | P value |
| --- | --- | --- | --- |
| n | 271 | 271 |  |
| Pathologic T stage, n (%) |  |  | 0.033 |
| T1&T2&T3 | 269 (49.6%) | 262 (48.3%) |  |
| T4 | 2 (0.4%) | 9 (1.7%) |  |
| Pathologic N stage, n (%) |  |  | 0.041 |
| N0 | 116 (45.8%) | 120 (47.4%) |  |
| N1 | 4 (1.6%) | 13 (5.1%) |  |
| Pathologic M stage, n (%) |  |  | 0.002 |
| M0 | 224 (44%) | 207 (40.7%) |  |
| M1 | 26 (5.1%) | 52 (10.2%) |  |
| Pathologic stage, n (%) |  |  | 0.002 |
| Stage I&Stage II&Stage III | 242 (44.9%) | 215 (39.9%) |  |
| Stage IV | 28 (5.2%) | 54 (10%) |  |
| Gender, n (%) |  |  | 0.015 |
| Female | 107 (19.7%) | 80 (14.8%) |  |
| Male | 164 (30.3%) | 191 (35.2%) |  |
| Age, n (%) |  |  | 0.048 |
| <= 60 | 147 (27.1%) | 124 (22.9%) |  |
| > 60 | 124 (22.9%) | 147 (27.1%) |  |
| Histologic grade, n (%) |  |  | 0.002 |
| G1&G2&G3 | 240 (45%) | 218 (40.9%) |  |
| G4 | 25 (4.7%) | 50 (9.4%) |  |
| OS event, n (%) |  |  | < 0.001 |
| Alive | 210 (38.7%) | 158 (29.2%) |  |
| Dead | 61 (11.3%) | 113 (20.8%) |  |
| DSS event, n (%) |  |  | < 0.001 |
| No | 235 (44.3%) | 187 (35.3%) |  |
| Yes | 32 (6%) | 76 (14.3%) |  |
| PFI event, n (%) |  |  | < 0.001 |
| No | 214 (39.5%) | 166 (30.6%) |  |
| Yes | 57 (10.5%) | 105 (19.4%) |  |

Supplementary Table 5 Relationship between hsa-miR-130b-3p expression and clinical characteristics in KIRC.

| Characteristics | Low expression of hsa-miR-138-5p | High expression of hsa-miR-138-5p | P value |
| --- | --- | --- | --- |
| n | 168 | 169 |  |
| Pathologic T stage, n (%) |  |  | 0.007 |
| T1&T2&T3 | 168 (49.9%) | 160 (47.5%) |  |
| T4 | 0 (0%) | 9 (2.7%) |  |
| Pathologic N stage, n (%) |  |  | 0.401 |
| N0 | 66 (42%) | 80 (51%) |  |
| N1 | 3 (1.9%) | 8 (5.1%) |  |
| Pathologic M stage, n (%) |  |  | 0.091 |
| M0 | 135 (42.7%) | 127 (40.2%) |  |
| M1 | 21 (6.6%) | 33 (10.4%) |  |
| Pathologic stage, n (%) |  |  | 0.035 |
| Stage I&Stage II&Stage III | 145 (43.3%) | 133 (39.7%) |  |
| Stage IV | 21 (6.3%) | 36 (10.7%) |  |
| Gender, n (%) |  |  | 0.758 |
| Female | 55 (16.3%) | 58 (17.2%) |  |
| Male | 113 (33.5%) | 111 (32.9%) |  |
| Age, n (%) |  |  | 0.624 |
| <= 60 | 86 (25.5%) | 82 (24.3%) |  |
| > 60 | 82 (24.3%) | 87 (25.8%) |  |
| Histologic grade, n (%) |  |  | < 0.001 |
| G1&G2&G3 | 156 (46.8%) | 117 (35.1%) |  |
| G4 | 10 (3%) | 50 (15%) |  |
| OS event, n (%) |  |  | < 0.001 |
| Alive | 130 (38.6%) | 100 (29.7%) |  |
| Dead | 38 (11.3%) | 69 (20.5%) |  |
| DSS event, n (%) |  |  | < 0.001 |
| No | 143 (43.1%) | 117 (35.2%) |  |
| Yes | 23 (6.9%) | 49 (14.8%) |  |
| PFI event, n (%) |  |  | 0.108 |
| No | 122 (36.2%) | 109 (32.3%) |  |
| Yes | 46 (13.6%) | 60 (17.8%) |  |

Supplementary Table 6 The relationship between hsa-miR-130b-3p and hsa-miR-138-5p expression and immune escape genes in KIRC

| gene | R(hsa-miR-130b-3p) | P(Spearman) | R(hsa-miR-138-5p) | P(Spearman) |
| --- | --- | --- | --- | --- |
| ADORA2A | -0.139 | 0.002 | -0.421 | <0.001 |
| BTLA | 0.136 | 0.002 | 0.083 | 0.14 |
| CD160 | -0.014 | 0.747 | -0.128 | 0.022 |
| CD274 | -0.12 | 0.007 | -0.042 | 0.457 |
| CD96 | 0.163 | <0.001 | 0.055 | 0.325 |
| CTLA4 | 0.215 | <0.001 | 0.115 | 0.039 |
| IDO1 | -0.104 | 0.019 | -0.208 | <0.001 |
| IL10 | 0.163 | <0.001 | 0.11 | 0.049 |
| IL10RB | -0.012 | 0.786 | -0.162 | 0.004 |
| KDR | -0.322 | <0.001 | -0.45 | <0.001 |
| KIR2DL1 | -0.058 | 0.189 | -0.217 | <0.001 |
| KIR2DL3 | 0.002 | 0.967 | -0.157 | 0.005 |
| LAG3 | 0.284 | <0.001 | 0.134 | 0.017 |
| PDCD1 | 0.231 | <0.001 | 0.11 | 0.05 |
| TGFB1 | 0.124 | 0.005 | -0.044 | 0.436 |
| TGFBR1 | -0.126 | 0.004 | -0.021 | 0.709 |
| TIGIT | 0.214 | <0.001 | 0.095 | 0.088 |


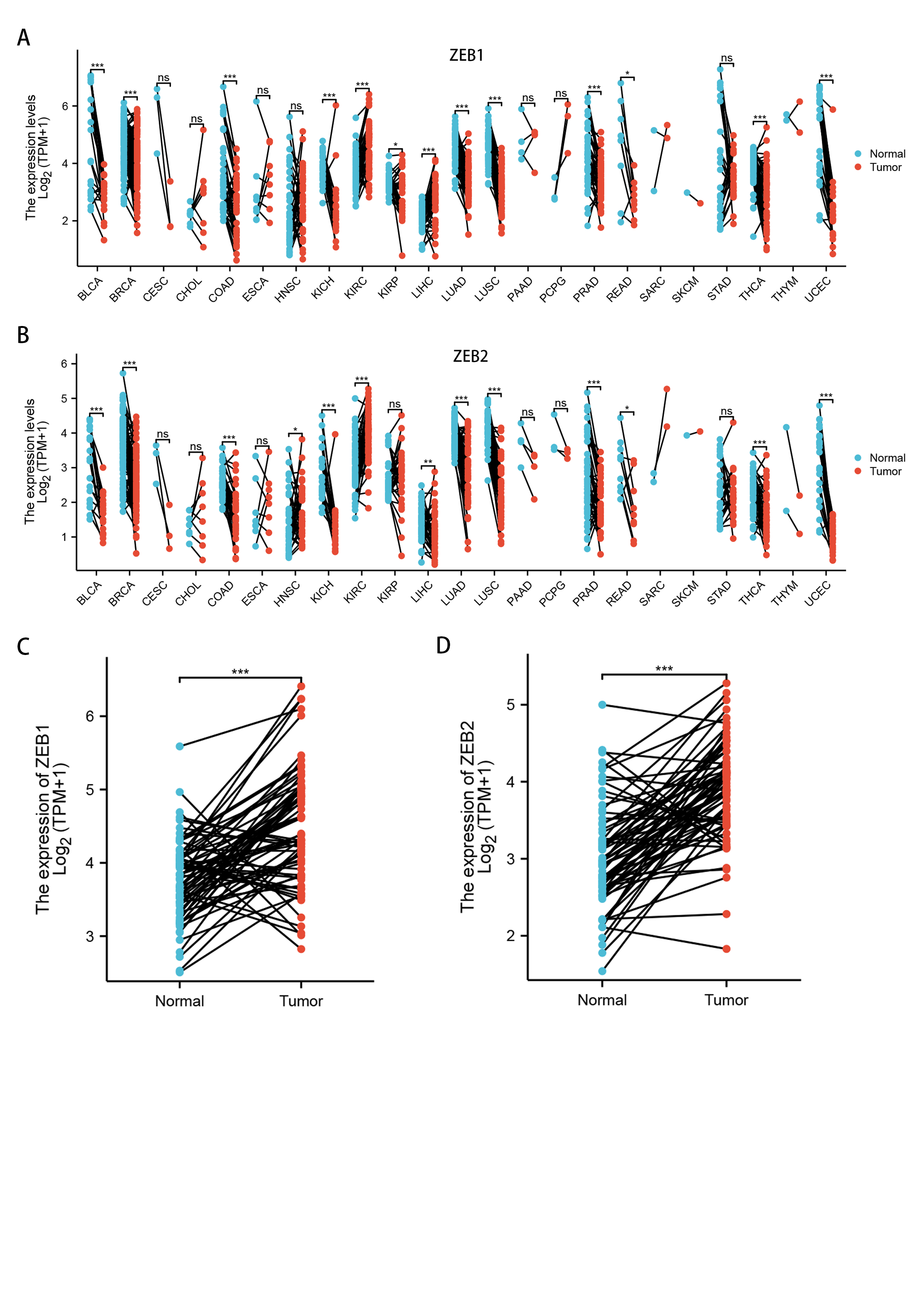
Supplementary Figure 1 The expressions of ZEB1 and ZEB2 in different type of cancer (A, B). And the expressions of ZEB1 and ZEB2 in KIRC (C, D).


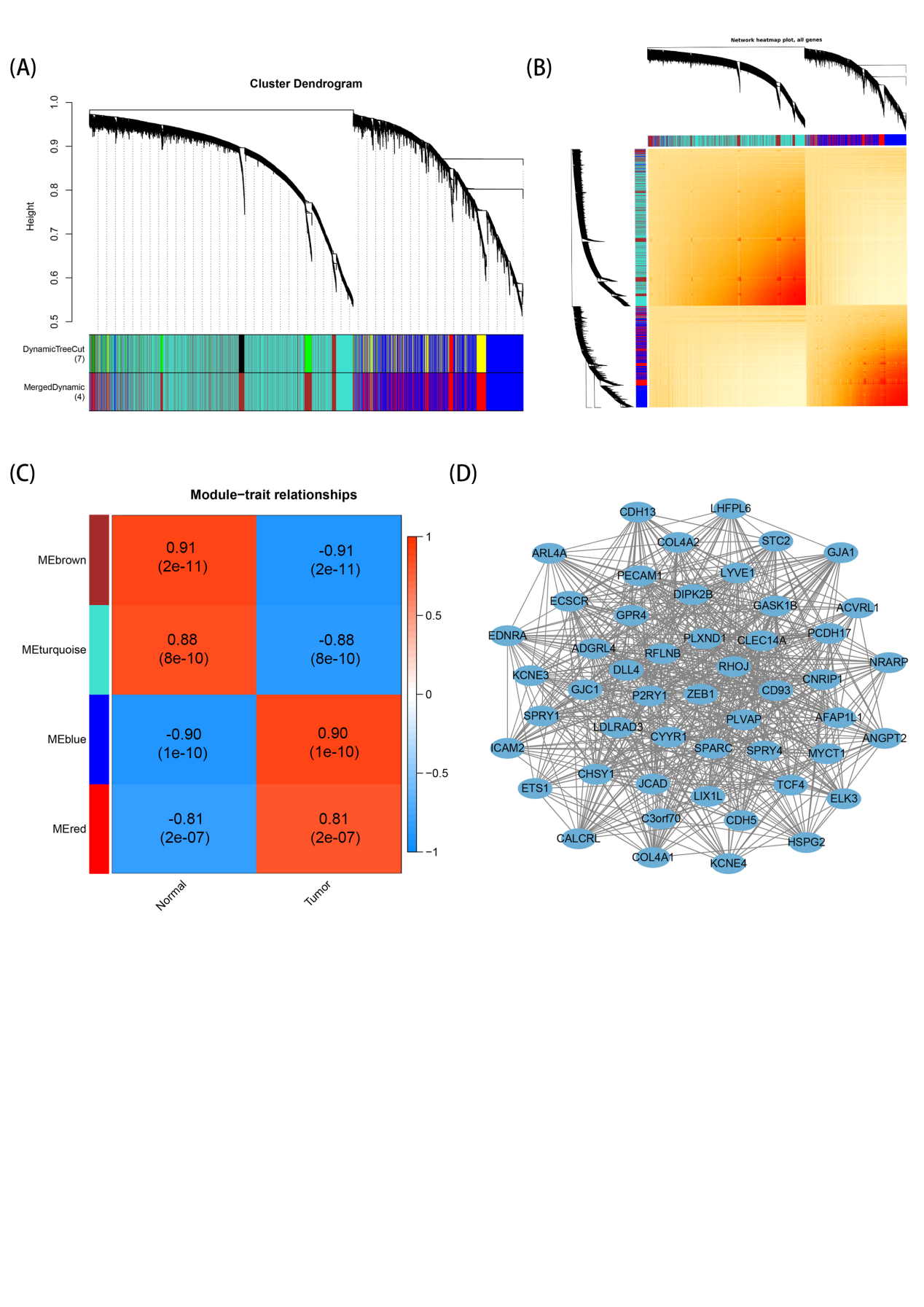


Supplementary Figure 2 The construction of WGCNA modules associated with ccRCC based on cohort GSE66270. (A) Cluster dendrogram of GSE66270 genes. Each branch represents a gene, and each color represents a co-expression module. (B) Network heatmap plots were generated for genes selected for WGCNA construction, with the depth of yellow indicating the degree of correlation between pair-wise genes. (C) Heatmap of the correlation between the module eigengenes and sample types of the GSE66270 cohort (ccRCC and normal kidney tissues). (D) The interaction network of the top 50 related genes in the red module.


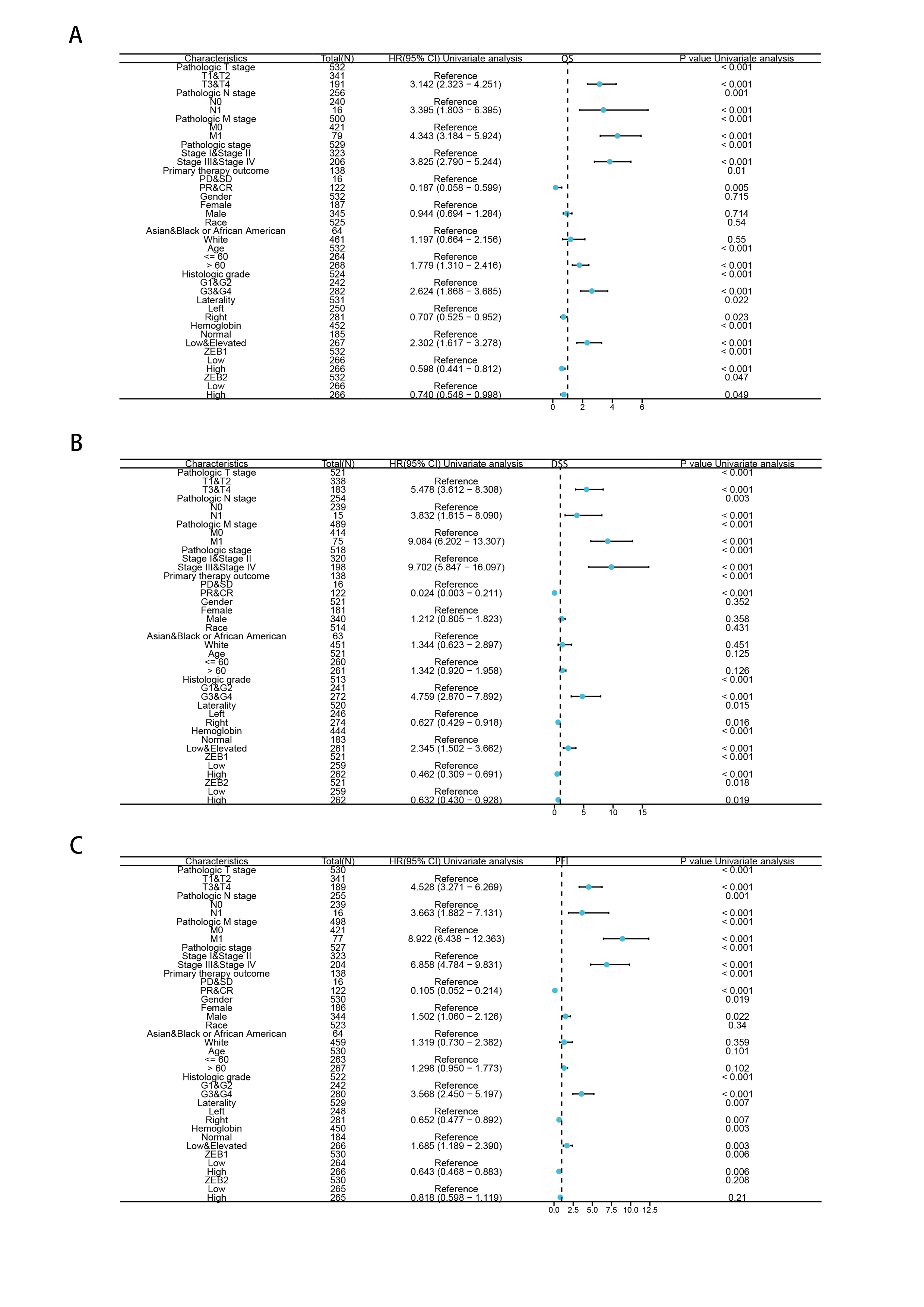
Supplementary Figure 3 Univariate Cox analysis of ZEB1, ZEB2 and different clinical variables for OS (A), DSS (B) and PFI (C) in KIRC.


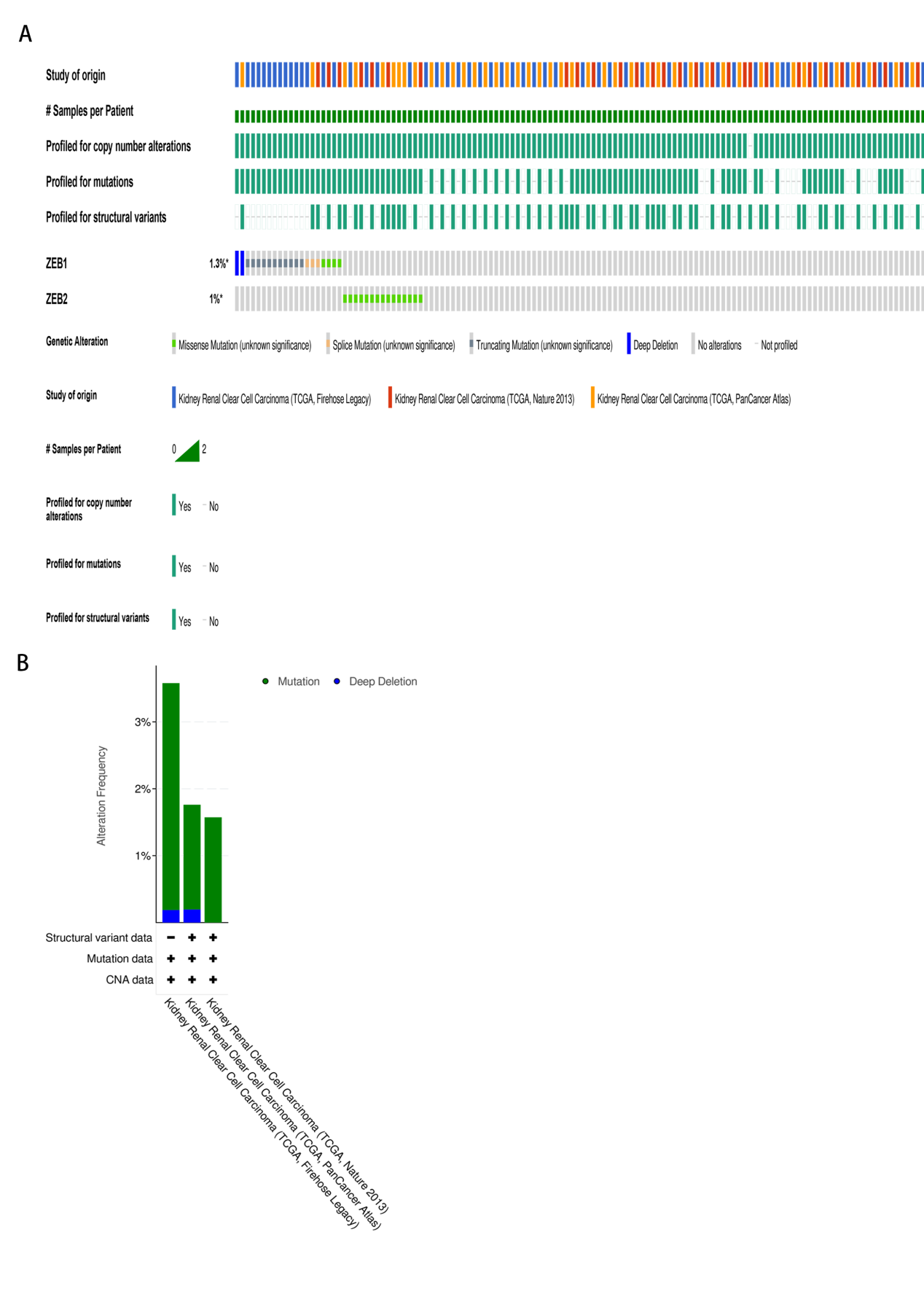
Supplementary Figure 4 Analysis of ZEB1 and ZEB2 mutations in KIRC. A, B, ZEB1 and ZEB2 mutations in 1496 KIRC patients from the cBioPortal database.


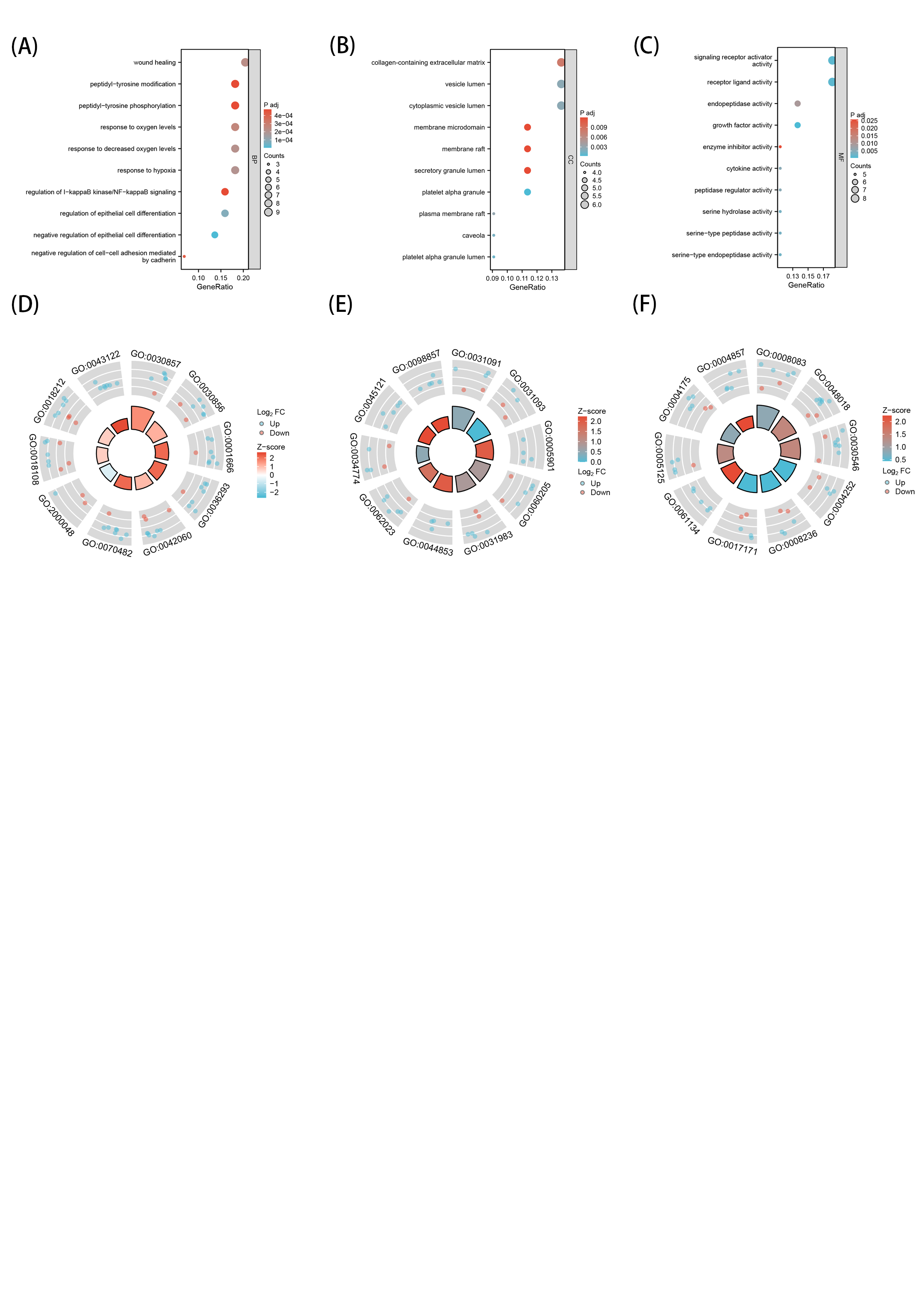
Supplementary Figure 5 The top 10 enriched GO terms of differentially expressed anoikis-related genes in BP(A), CC(B) and MF(C) categories in KIRC.


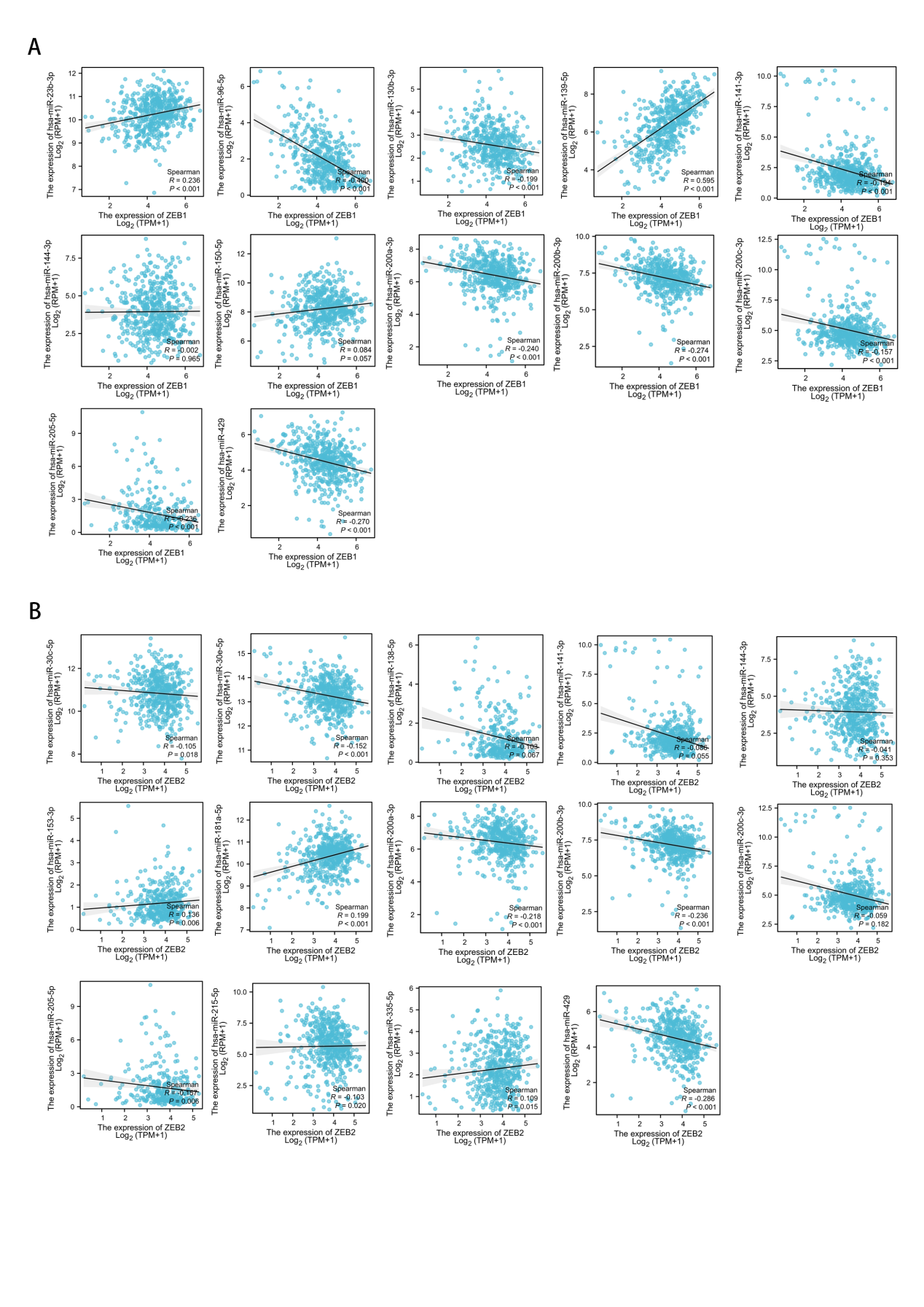
Supplementary Figure 6 Scatter plots of the correlations between ZEB1 (A) and ZEB2 (B) expression and microRNAs targeting ZEB1 or ZEB2 in KIRC.


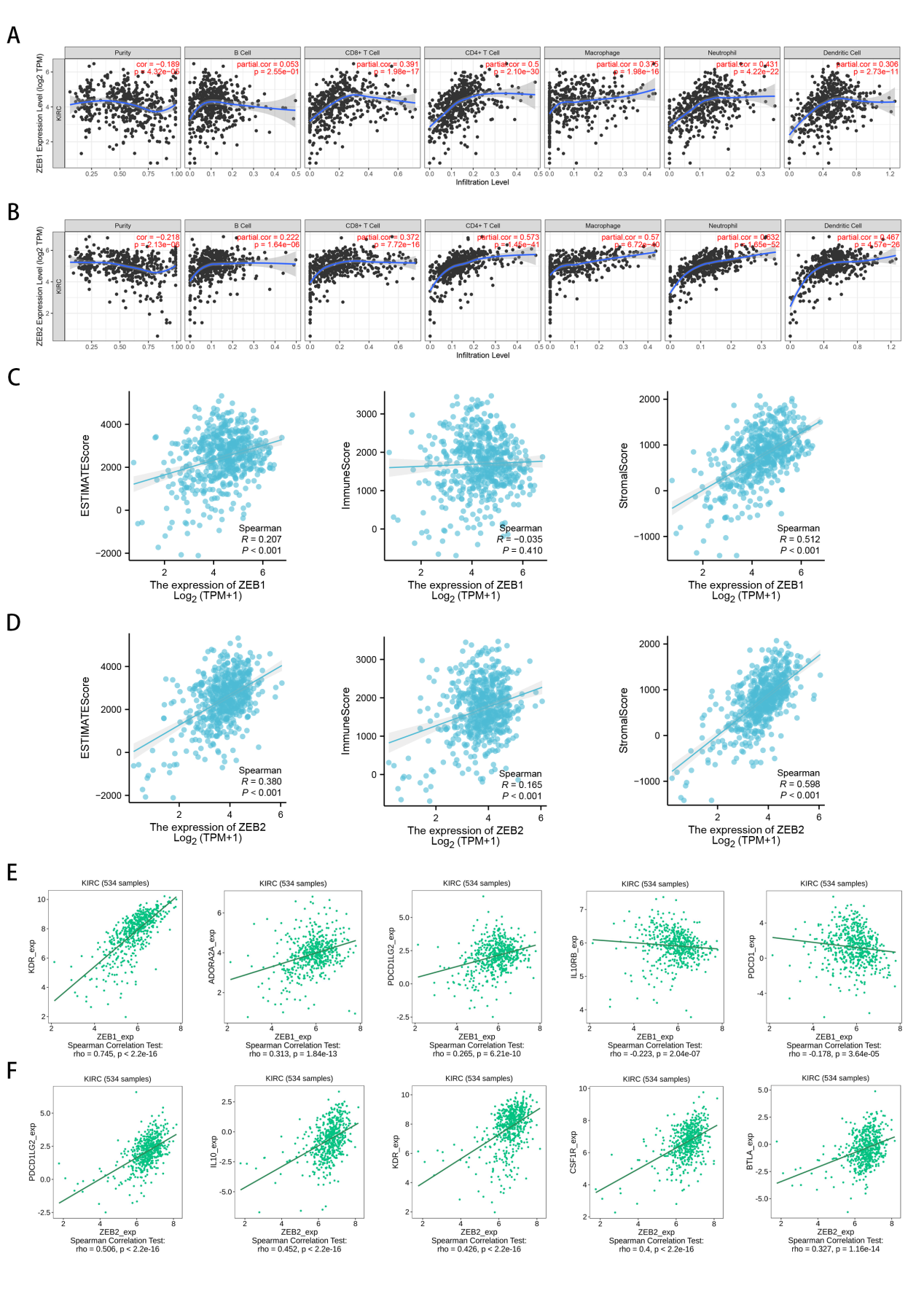
Supplementary Figure 7 The correlations of ZEB1, ZEB2 and immune infiltration in KIRC tissues by using TCGA dataset. A, Using TIMER2.0 database analysed the correlation of ZEB1 and different immune cell infiltration. B, The correlation of ZEB2 and different immune cell infiltration. C, D, The correlation of ZEB1 and ZEB2 expression level with ESTIMATE score, immune score, as well as stromal score. E, Using TISIDB dataset analysed the correlations of ZEB1 expression and different genes (KDR, ADORA2A, PDCD1L, IL10RB and PDCD1). F, The correlations of ZEB2 expression and different genes (PDCD1LG2, IL10, KDR, CSF1R and BTLA).
